# Repurposing Ketamine in the Therapy of Depression and Depression-Related Disorders: Recent Advances and Future Potential

**DOI:** 10.14336/AD.2024.0239

**Published:** 2024-04-29

**Authors:** Qianting Deng, Emily Parker, Chongyun Wu, Ling Zhu, Timon Cheng-Yi Liu, Rui Duan, Luodan Yang

**Affiliations:** ^1^College of Physical Education and Sport Science, South China Normal University, Guangzhou, China.; ^2^Medical College of Georgia at Augusta University, Augusta, GA 30912, USA.

**Keywords:** depression, ketamine, antidepression, neuropsychiatric disorders, ketamine repurposing

## Abstract

Depression represents a prevalent and enduring mental disorder of significant concern within the clinical domain. Extensive research indicates that depression is very complex, with many interconnected pathways involved. Most research related to depression focuses on monoamines, neurotrophic factors, the hypothalamic-pituitary-adrenal axis, tryptophan metabolism, energy metabolism, mitochondrial function, the gut-brain axis, glial cell-mediated inflammation, myelination, homeostasis, and brain neural networks. However, recently, Ketamine, an ionotropic N-methyl-D-aspartate (NMDA) receptor antagonist, has been discovered to have rapid antidepressant effects in patients, leading to novel and successful treatment approaches for mood disorders. This review aims to summarize the latest findings and insights into various signaling pathways and systems observed in depression patients and animal models, providing a more comprehensive view of the neurobiology of anxious-depressive-like behavior. Specifically, it highlights the key mechanisms of ketamine as a rapid-acting antidepressant, aiming to enhance the treatment of neuropsychiatric disorders. Moreover, we discuss the potential of ketamine as a prophylactic or therapeutic intervention for stress-related psychiatric disorders.

## 1. Introduction

Depression, a ubiquitous mental ailment afflicting individuals worldwide, poses a significant public health concern [1]. Boasting a staggering global prevalence of approximately 264 million individuals across all age groups, depression is anticipated to burgeon into the second foremost cause of global disease and disability by 2030, as stipulated by the World Health Organization [1, 2]. Moreover, compelling evidence highlights the dangerous susceptibility of individuals with severe depressive disorders to develop suicidal tendencies [1]. Although organisms possess mechanisms to counterbalance the potentially harmful side effects of short-term stress responses, prolonged exposure to stress gives rise to unfavorable physiological and behavioral alterations [3].

The main features of depression include melancholic mood, anhedonia, fatigue, difficulty concentrating, sleep problems, changes in appetite, cognitive function, and self-harm tendencies [4]. Regrettably, the intricate molecular mechanisms that underlie the etiopathogenesis of depression remain incompletely elucidated [5]. Major systems under consideration include monoamines, excitatory and inhibitory neurotransmission, neurotrophins, mitochondrial dysfunction, glial cell impairment, the hypothalamic pituitary adrenal (HPA) axis hyperactivity, tryptophan (TRP) metabolism disruption, demyelination, and dysbiosis, among others. Anxiety and depressive disorders frequently coexist, with up to 85% of individuals diagnosed with depressive disorders concurrently experiencing anxiety disorders [6]. Depression not only exhibits a high rate of comorbidity with anxiety disorders, but it also demonstrates close associations with other brain disorders and other diseases [7-14]. These diseases include post-stroke depression (PSD); post-traumatic stress disorder (PTSD); Alzheimer's disease (AD); Parkinson’s disease (PD); Huntington’s disease (HD); Type 2 diabetes (T2D); Multiple sclerosis (MS); and inflammatory bowel disease (IBD) [7-14] (Table 1). However, the chronic and debilitating nature of depression complicates the prognosis of numerous chronic diseases, which further exacerbates the global landscape of disease burden and mortality [4].

**Table 1:** Mechanisms and pathological changes disorders associated with depression.

Disease	Mechanisms	Pathological changes	Ref.
**PSD**	Microglia-mediated neuroinflammation	Decreased miR34b-3p and increased eIF4E	[247]
	The mediodorsal thalamic nucleus hyperactivity	Parvalbumin-positive interneurons hyperactivate impaired the local excitatory and inhibitory balance within the prefrontal cortical microcircuitry	[249]
	HPA axis dysregulation	Increased GR sensitivity and negative feedback inhibition of the HPA axis	[252]
	Glutamatergic systems	Glutamatergic synaptic strength disruption	[255]
**AD**	Aβ peptides	Disrupts serotoninergic functions (5-HT and NE) and neurotrophic factor signaling	[263]
	HPA axis dysregulation	Increased Glucocorticoid levels and elevated cellular injury and apoptosis	[269]
	CALHM family proteins	CALHM2 V136G mutation disrupted ATP release	[9]
PD	PF→NAc circuit	Aberrant connectivity between anxiety-depression pathways and basal ganglia network activity	[282]
**HD**	Cdk5/DARPP-32/β-adducin signaling pathway	Disturbed the dendritic spine cytoskeleton	[11]
	Astrocyte-mediated K^+^ homeostasis	Excessive K+ and increased medium spiny neurons excitability	[292]
T2D	HPA axis hyperactivation	Increased cortisol levels and decreased insulin sensitivity	[12]
	Inflammatory cytokines	TRP into neuroactive metabolites; Increased serotonin levels	[12]
	Insulin signaling disruption	Decreased dopamine release and astrocytes mediated- ATP release	[298]
	Microglial NLRP3	Increased P2X7R, ROS production, and TXNIP expression	[302]
MS	Myelin impairment gut microbiota alterations	Aberrant communications between integral anatomical regions implicated in task-specific emotion regulation	[109, 310]
**Osteoporosis**	GABAergic neural circuity and BNST-VMH-NTS neural circuitry	Increased somatostatin neurons activation and decreased SF-1 neurons activity	[320]
	OPG-RANK-RANKL system and OPN	Decreased OPG/RANKL, plasma OPN level, and BMD; Elevated RANKL	[322]
**IBD**	Gut-brain-vascular axis dysregulation	Disruption of the GVB coincides with bacterial product translocation and systemic inflammation	[329]
	Gut microbiota	Increased Deltaproteobacteria levels	[234]
	Microglial TREM-1 and TREM-2 receptor imbalance	Abnormalities in the modulation of glutamatergic neurons	[332]
	Inflammatory chemokines	The upregulation of Lcn2 causes the loss of dendritic spines and secreted proteins	[333]
	Microbial dysregulation	Estrogen receptor β disrupted neural processing within the gut-brain axis	[335]
	TRP metabolism disturbance	Increased quinolinic acid	[337]

PSD: post-stroke depression; PTSD: post-traumatic stress disorder; AD: Alzheimer's disease; PD: Parkinson’s disease; HD: Huntington’s disease; T2D: Type 2 diabetes; MS: Multiple sclerosis; IBD: Inflammatory bowel disease

Presently, the most used antidepressants are selective serotonin reuptake inhibitors and serotonin-norepinephrine reuptake inhibitors [15]. However, these drugs are subject to several limitations, including delayed therapeutic response rates, treatment resistance in approximately 30% of patients, and mitigation failure for the heightened propensity for suicide [15]. Notedly, the heightened risk of suicide among depressed individuals is commonly attributable to the delayed onset of antidepressant effectiveness [16]. Hence, it is imperative for therapies to not only alleviate depressive symptoms but also promptly diminish suicidal ideation [17].

Ketamine was initially developed in the 1970s for anesthesia and analgesia [18]. Over several decades of its use as an anesthetic and analgesic, and in unsupervised illicit use, ketamine has been associated with perceptual disturbances, emergence of delirium, dissociative symptoms, and potential abuse [18]. However, a groundbreaking study observed a significant reduction in depressive symptoms within 4 hours of ketamine treatment [19]. Numerous studies have shown that ketamine offers robust and enduring efficacy for depression [17, 20, 21]. Despite a reduced dosing frequency, improvements in depressive symptoms persisted during the open-label phase and continued up to 2 months after discontinuation of ketamine [22]. It is believed that ketamine may mitigate synaptic loss induced by stress, thereby broadly alleviating depression and restoring neuronal microcircuit connectivity [23]. Based on these favorable results, the FDA approved (S)-ketamine as an adjunctive treatment for depressive disorder [24]. Nevertheless, the approval of (S)-ketamine and its application in depression therapy has sparked controversy due to concerns about adverse reactions, such as potential abuse and the onset of psychotomimetic effects [25]. In response, these controversies have spurred research into the cellular and molecular mechanisms underlying the antidepressant effects and adverse reactions of ketamine. Although the discovery of ketamine demonstrates promise as a treatment for depression, further exploration is required to elucidate mechanisms and drug repurposing in other diseases. Therefore, this review discusses antidepressant mechanisms of ketamine and its emerging therapeutic applications.

**Figure 1.**
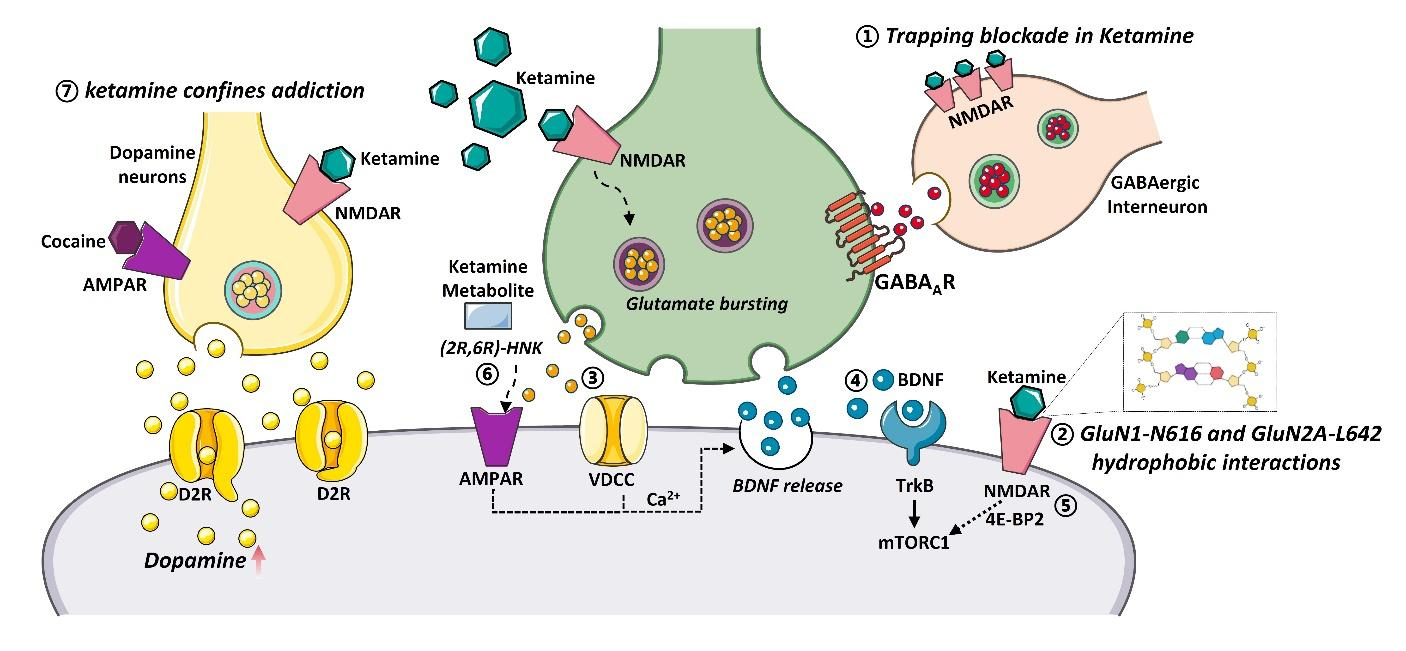
**The basal properties of antidepressant actions of ketamine**. (1) Ketamine belongs to a unique class of use-dependent trapping blockers, resulting in a prolonged antidepressant effect; (2) hydrogen bonding at GluN1-N616 and hydrophobic interactions at GluN2A-L642 facilitate blockade of the channel; (3) Ketamine inhibited NMDAR produces a rapid glutamate burst acting on AMPAR, leading to the opening of VDCC that stimulate BDNF release; (4) BDNF binding to TrkB orchestrates the formation of the mTORC1, regulating protein synthesis and cell proliferation; (5) the mTORC1 downstream molecular 4E-BP2 mediated synaptic transmission; (6) ketamine-induced antidepressant efficacy also proposed non-NMDAR-dependent mechanisms; (7) ketamine did not elicit drug-induced synaptic plasticity, locomotor sensitization, or uncontrolled self-administration.

## 2. The basal properties of antidepressant actions of ketamine

Ketamine, an antagonist of the N-methyl-D-aspartate receptor (NMDAR), provides a promising opportunity to develop better treatment of depression because of its rapid and sustained antidepressant effects [26]. Intriguingly, while the elimination half-life of ketamine in mice is only three hours, its antidepressant activity in humans lasts up to one week [27]. Recent research ascertains that ketamine belongs to a unique class of use-dependent trapping blockers, characterized by a collection of biophysical mechanisms that are commonly shared between its rapid and prolonged antidepressant effects (Fig. 1) [27]. By selectively obstructing NMDARs in the open state and subsequently being trapped within the channel pore to prevent swift metabolism, ketamine achieves persistent inhibition of downstream neural bursting, resulting in a prolonged antidepressant effect [27]. Therefore, ketamine binding to glutamate receptors is responsible for rapid antidepressant activity, while ketamine's unique trapping in the receptor is necessary for long-lasting antidepressant properties [27]. Furthermore, another piece of evidence has underscored the crucial roles of hydrogen bonding at GluN1-N616 and hydrophobic interactions at GluN2A-L642, collectively stabilizing ketamine binding to the channel pore of the NMDARs and facilitating blockade of the channel [28].

The antidepressant effect of ketamine is initiated by the selective blockade of NMDAR-mediated inhibition of γ-aminobutyric acid (GABA) interneurons [29]. In turn, disinhibition of neuronal activity and pyramidal neurons leads to enhanced glutamatergic firing, then stimulates postsynaptic alpha-amino-3-hydroxy-5-methyl-4-isoxazolepropionic acid receptors (AMPARs), leading to depolarization and activation of L-type voltage-dependent Ca^2+^ channels (VDCCs) and subsequent release of brain-derived neurotrophic factor (BDNF) [29]. BDNF binding to tropomyosin receptor kinase B (TrkB) orchestrates the formation of the mechanistic target of rapamycin complex 1 (mTORC1), regulating protein synthesis and cell proliferation [29]. Notedly, further investigation suggests that ketamine triggers antidepressant effects through the recruitment of the mTORC1 downstream molecular, eukaryotic translation initiation factor 4E-binding protein 2 (4E-BP2), mediated synaptic transmission within the hippocampus [30]. In addition, the sustained actions of ketamine-induced antidepressant efficacy also suggest non-NMDAR-dependent mechanisms [31]. Ketamine undergoes diverse metabolic transformations in the brain, with (2R,6R)-hydroxynorketamine (HNK) and (2S,6S)-HNK being the principal metabolites [32]. The metabolism of ketamine to (2R,6R)-HNK is crucial for its antidepressant effects and is associated with sustained activation of AMPAR, enhancing excitatory synapses in emotion-related brain regions [30]. However, (2R,6R)-HNK enhances AMPAR activity and directly interacts with the TrkB, (2S,6S)-HNK exerts minimal effects in this regard [30, 32]. Consequently, (2S,6S)-HNK and (2R,6R)-HNK may possess distinct mechanisms of action [33]. Chronic stress induces diminished neuronal activity in the anterior paraventricular nucleus and posterior paraventricular nucleus of the thalamus [33]. Recent investigations have revealed that (2S,6S)-HNK transiently activates glutamatergic neurons in the anterior paraventricular nucleus, leading to the transcriptional upregulation of Gabra4, Gabrb2, and Gabrd genes within 6 hours [33]. Subsequently, in three days later, (2S,6S)-HNK-mediated induction of GABA_A_Rs enhances tonic inhibition of glutamatergic neurons in the anterior paraventricular nucleus [33].

Additionally, the reduction of hippocampal neurogenesis is observed in patients with depression, while antidepressant medication promotes neurogenesis [34]. Adult neurogenesis primarily occurs in the dentate gyrus (DG) and subventricular region [35]. The differentiation of hippocampal neural stem cells into mature granule cells and their integration into existing hippocampal neural circuits takes several weeks, and a time frame believed to contribute to the delayed onset of action for most antidepressants [35, 36]. Ketamine relies on hippocampal AMPAR activation to exert rapid antidepressant effects on the adult-born immature granule neurons in the mouse hippocampal DG region [37]. Meanwhile, ketamine also facilitates hippocampal neurogenesis, synaptic plasticity, and increased expression of brain-derived neurotrophic factors, exerting rapid antidepressant effects [37]. This may account for ketamine's ability to produce antidepressant effects within hours, unlike classical antidepressants, which often take weeks to exert their effects [37]. Other downstream molecules and cellular pathways have also been investigated to elucidate the rapid antidepressant properties of ketamine and its role in facilitating neuroplasticity [38]. For instance, extracellular signal-regulated kinase is involved in the antidepressant actions of ketamine [38]. Hence, integrating multiple pathways may provide the most comprehensive understanding of the distinct therapeutic effects of ketamine.

Given the increasing utilization of ketamine as a rapid-acting antidepressive agent, the debate regarding its potential for psychotic side effects and addiction risk has resurfaced. Studies in rodents suggest that ketamine carries a risk of addiction, as early experiments indicate that ketamine administration produces cocaine-like behaviors, including a general increase in dopamine in the nucleus accumbens (NAc) and changes in reward and reinforcement behaviors [39, 40]. However, recent studies have found that ketamine causes transient fluctuations in dopamine levels in the NAc, enhancing reward effects by disinhibiting dopamine neurons in the ventral tegmental area (VTA) [41]. Still, it does not induce addiction-related mechanisms [41]. Ketamine exerts its rewarding effects by inhibiting NMDAR activity on inhibitory neurons in the VTA, promoting dopamine neuron activity [41]. Subsequently, the activation of the dopamine neurons induces a short burst of dopamine released in NAc and then rapidly ceases upon dopamine binding to the D2-type receptor (D2R) protein on the dopamine neurons, preventing excessive release of dopamine [41]. Consequently, unlike addictive drugs such as cocaine, ketamine does not elicit drug-induced synaptic plasticity, locomotor sensitization, or uncontrolled self-administration [41].

Similarly, different subtypes of NMDAR exhibit distinct expression patterns and electrophysiological characteristics, which may contribute differently to the antidepressant effects and side effects of ketamine [42]. NMDARs, predominantly composed of GluN2A and GluN2B subunits, have been implicated in the antidepressant response to ketamine [43]. GluN2B is widely expressed in GABAergic interneurons and glutamatergic principal neurons [43]. Previously, GluN2B was considered one of the direct targets of ketamine's antidepressant response [44]. Ketamine induces rapid protein synthesis, enhances communication between cortical neurons, and mitigates depression through GluN2B-containing signaling [43]. Unfortunately, no GluN2B-selective inhibitors have been approved for clinical use [44]. Interestingly, studies have shown that removing GluN2A from the hippocampus is sufficient to induce antidepressant-like behavior [15]. Additionally, GluN2A inhibition suppresses intrinsic excitability in CA1 principal neurons, while selective inhibition of GluN2B is ineffective in altering neuronal excitability [15]. Therefore, it is speculated that GluN2A in excitatory neurons also mediates ketamine-induced rapid antidepressant-like responses, possibly with fewer side effects through mechanisms distinct from GluN2B [15]. Although everyday adverse events associated with intranasal esketamine include hallucinations, hypertension, tachycardia, and vestibular symptoms, the emergence of ketamine may provide the ability to understand the actual core pathogenic mechanism of depression [29]. Based on current knowledge, further investigation could develop more effective drugs with fewer side effects. Meanwhile, the long-term risks and side effects of ketamine and esketamine for depression are not well described and require more evidence to understand the safety of these drugs better [29]. Therefore, exploring multiple mechanisms of ketamine’s antidepressant effects is increasingly necessary to guide the development of safer and faster-acting antidepressants.

## 3. The pathological mechanism of depression and the antidepressant response to ketamine

### 3.1. The monoamine theory and neurotransmitters

One of the major hypotheses in the pathophysiology of depression is the monoamine hypothesis, which posits that biological mechanisms underlying depression are the alterations in monoamine levels, i.e., serotonin (5-HT), norepinephrine (NE), and dopamine (DA) [45]. This "monoamine theory of depression" has received preliminary support, as evidenced by reduced 5-HT metabolites and NE activity in patients diagnosed with depressive disorder [46].

*Norepinephrine.* It has been postulated that the noradrenergic system, closely intertwined with the neuroendocrine and immune systems, undergoes modifications in response to chronic stress [47]. Repeated exposure to emotional stress facilitates NE imbalance within the brain, particularly leading to heightened responsiveness of neurons in the locus coeruleus [48, 49]. Stress prompts a hyperactivation of noradrenergic neurons, leading to the diminished activation of α2-receptors at the presynaptic terminal, which projects to the locus coeruleus neurons, ultimately culminating in decreased levels of NE release [50]. Notably, dopamine-β-hydroxylase (DβH), a crucial enzyme involved in mood disorders, represents a rate-limiting step in NE biosynthesis, acting as one of the monoamine pathway enzymes responsible for converting DA into NE [51]. Recently, a study suggested that chronic stress or prolonged inflammatory stimulation may increase lipopolysaccharide-binding protein levels, inhibiting DβH activity and reducing NE levels [49].

**Figure 2.**
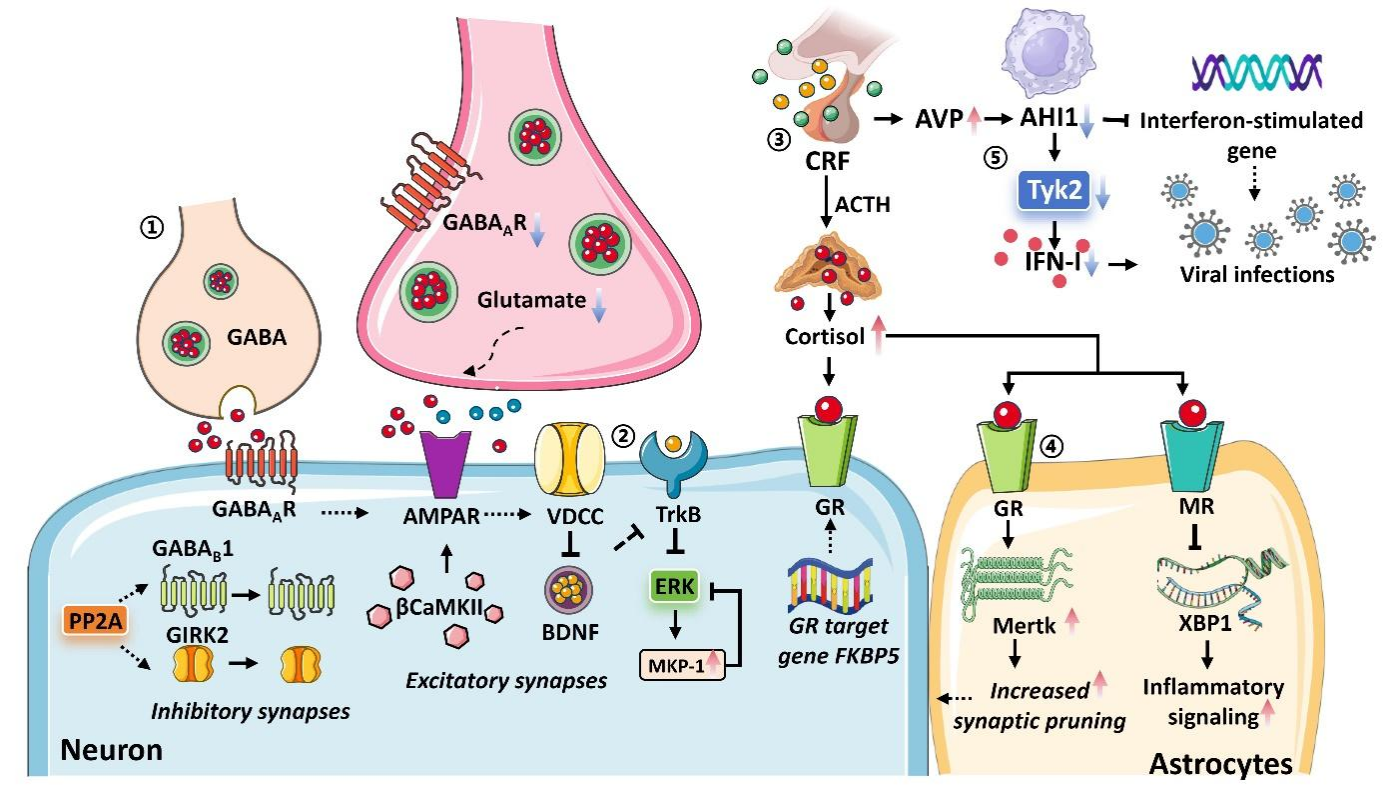
**The pathological changes of depression in the dopaminergic System**. (1) Stress prompts a hyperactivation of noradrenergic neurons, leading to the diminished activation of α2-receptors, ultimately culminating in lowered levels of NE release. Furthermore, DβH represents a rate-limiting step in NE biosynthesis; (2) Chronic restraint stress decreases neuronal activity in D3 neurons, inhibiting synaptic connections with spiny projection neurons and reducing dopamine release. Additionally, reduced excitatory input in dopamine D1R have been causally linked to stress-induced depressive-like behaviors; (3) Kir4.1 upregulation cause a decrease in extracellular K^+^ and hyperpolarization of neurons; (4) Chronic stress induces myelin loss through the Wnt/β-catenin signaling pathway and D2R-mediated DA signaling; (5) Increase in EphA4 protein causes demyelination and synaptic malfunctions; (6) Social adversity induces neuronal mitochondrial fission through Drp1 mediation, which has a harmful impact on local ATP synthesis and impinges on neuronal AMPAR-dependent synaptic transmission; (7) Mitochondrial impairment activates the PINK1-Parkin mitophagy pathway, leading to excessive mitochondrial elimination and weakening anxiolytic pathway; (8) diminished levels of MFN2 inducing the reduction of mitochondrial GTPase; (9) NHE1 deficits attributed the ubiquitination and degradation of NHE1 by activating E3 ubiquitin ligase cullin4A, ultimately leading to intracellular acidification.

*Dopaminergic System.* Dopamine is the principal neurotransmitter in the brain's extrapyramidal system and a precursor to adrenaline and noradrenaline, which are crucial in regulating behavior [52]. Increasing evidence suggests a close association between depression and dopaminergic transmission in the central nervous system (CNS) (Fig. 2) [53, 54]. The ventral striatum is a key reward center that integrates inputs from the entire brain, including midbrain dopaminergic neurons, and sends inhibitory outputs to downstream structures [55]. Within the ventral striatum lies the Calleja island, a cluster of granular cells that express dopamine D3 receptors and is primarily located in the olfactory tubercle (OT) [53]. In mice subjected to chronic restraint stress, decreased neuronal activity in OT D3 neurons was observed to inhibit synaptic connections with spiny projection neurons, significantly suppressing VTA dopaminergic neurons projecting to the NAc and reducing dopamine release, ultimately inducing depressive-like behavior [53]. Notedly, MSNs constitute the predominant cell type in the NAc and serve as the primary projection neurons, have been implicated in the manifestation of motivational deficits in animal models of depression [56]. Specifically, reduced excitatory input and dendritic complexity in NAc MSNs expressing dopamine D1 receptors (Drd1) have been causally linked to stress-induced depressive-like behaviors [57]. Preclinical rodent studies suggest that acute stress induces glutamate and dopamine release in the mPFC, promoting dendritic arborization and spine formation in mPFC pyramidal neurons through Drd1 activation while reducing stress-induced behavioral deficits [58]. Nevertheless, the mechanisms underlying the activation of dopamine neurons by ketamine remain ambiguous [59]. Involvement of Drd1 enhances the expression of surface NMDARs and AMPARs, thereby facilitating increased excitability and synaptic inputs in mPFC pyramidal neurons, potentially contributing to the promotion and strengthening of ketamine-induced synaptic connections [59].

Acute ketamine administration, through its NMDAR antagonistic action, has been found to broadly impact the dopaminergic regulatory system, resulting in the increased firing of dopamine neurons in the VTA and enhanced dopamine release in the PFC, striatum and NAc [41, 60, 61]. Repeated ketamine use leads to structural changes in the brain's dopamine system and alters dopamine neuron projections to the PFC and sensory areas [61]. Chronic ketamine exposure reduces dopamine neurons in behaviorally relevant midbrain regions but increases dopamine neurons in the hypothalamus [61]. Hypothalamic dopamine neurons regulate essential bodily functions, suggesting ketamine's potential in managing eating disorders [61]. Ketamine also alters dopamine fiber density in various brain regions, leading to dissociative effects [61]. Thus, repetitive ketamine treatment needs to target specific brain regions to minimize unintended effects on other dopamine areas.

**Figure 3.**
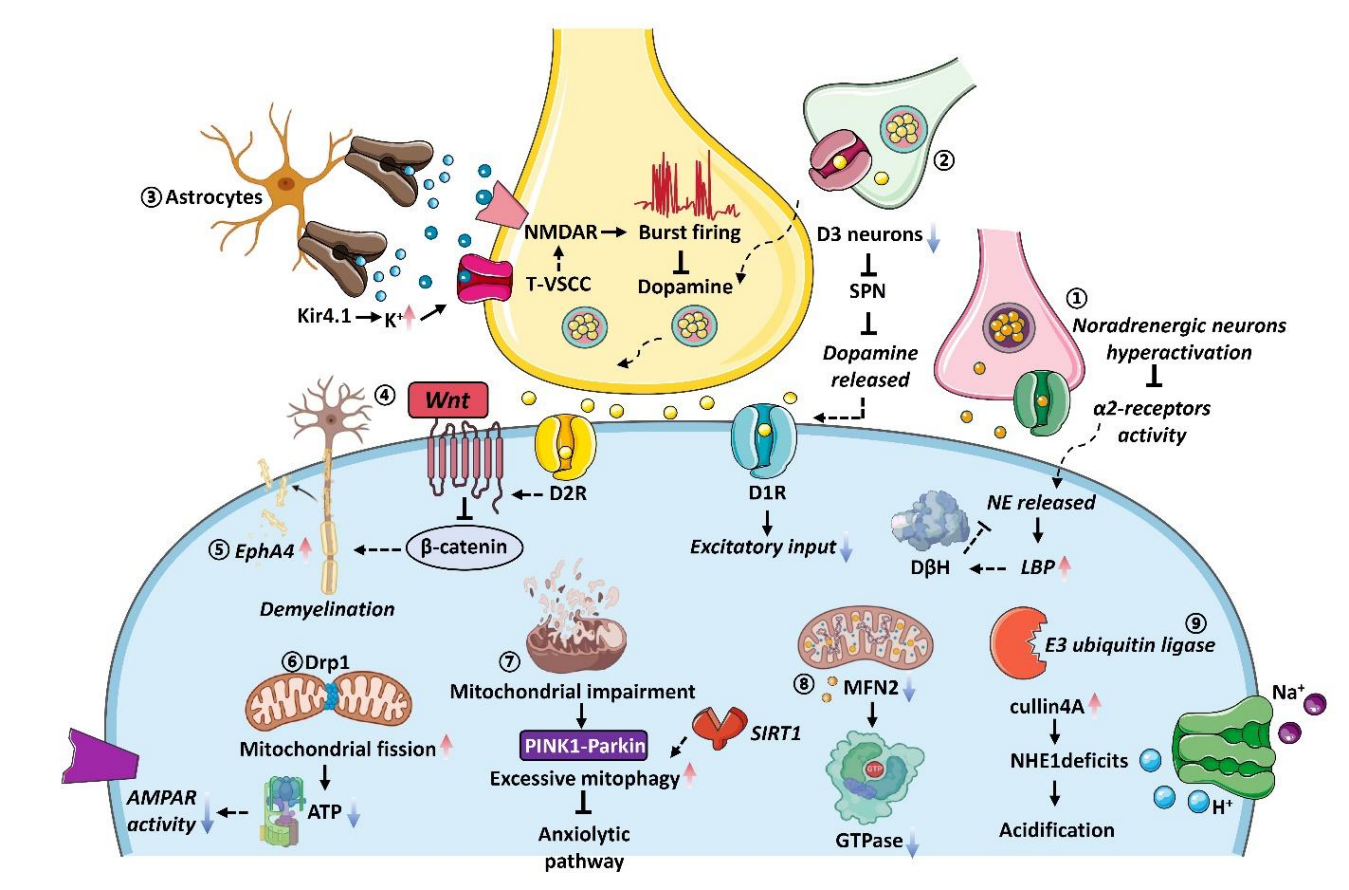
**The pathological mechanism of depression in glutamatergic systems and GABA**. (1) Stress and depression disrupt GABA neurotransmitter systems, activation of PP2A triggers the internalization of GABAB1 and GIRK2, leading to an augmentation in neuronal excitability, and upregulation of βCaMKII facilitates the translocation of AMPARs to the synaptic membrane, resulting in enhanced synaptic efficacy; (2) stress-induced downregulation of BDNF expression and reduced TrkB-mTORC1 signaling; (3) chronic stress exhibit high cortisol secretion, leading to the high expression of the GR, and GR target gene FKBP5 is also a significant player in depression; (4) cortisol triggers the expression of the GR in astrocytes driving the engulfment receptor Mertk, and the MR expression in astrocytes, which confines the activation of XBP1 that fosters pro-inflammatory signaling; (5) abnormally heightened AVP levels lead to a decline in macrophage AHI1 expression, diminishing the efficiency of interferon-mediated antiviral innate immunity.

*Glutamatergic systems and GABA.* Previously, antidepressant medications, including tricyclic antidepressants (TCA), SSRI, monoamine oxidase (MAOA) inhibitors, and SNRI, have been shown to contribute to an elevation in brain monoamine levels [62]. However, the clinical effects of antidepressant treatment are typically observed after several weeks, while the increase in monoamine levels by antidepressant drugs is almost instantaneous [46]. Moreover, monoamine deficiency may not be consistent across all patients with depressive disorder, indicating the involvement of alternative pathways in its pathogenesis, such as glutamatergic system and gamma-aminobutyric acid (GABA) [46, 63]. Glutamate and GABA are the major excitatory and inhibitory neurotransmitters, respectively, regulating both intrinsic and extrinsic modulation of cerebral information transmission [63]. The reduction levels of glutamate in specific brain regions of depressed patients have been well-documented in numerous studies [64-66]. Compelling evidence supports that glutamate is associated with reduced response to emotional stimuli [67]and decreased synaptic density and dendritic formation [68, 69]. Similarly, stress and depression perturb the GABAergic neurotransmitter systems, the predominant inhibitory pathways that modulate and refine excitatory signal transmission [63]. Furthermore, reduced levels of GABA, GABA-synthesizing enzymes, and neuropeptides have been detected in the cerebrospinal fluid, medial PFC, and various cortical brain regions among individuals diagnosed with depression [70, 71]. At the postsynaptic terminals of excitatory synapses, stress-induced upregulation of βCaMKII facilitates the translocation of AMPARs to the synaptic membrane, resulting in enhanced synaptic efficacy [72]. Similarly, at the postsynaptic terminals of inhibitory synapses, stress-induced activation of PP2A activation initiates the internalization of GABAB1 and GIRK2, ultimately resulting in increased neuronal excitability (Fig. 3) [72].

In contrast, ketamine administration promptly enhances the GABA and glutamate systems, thereby rectifying deficits arising from prolonged exposure to stress [71]. Preclinical investigations have demonstrated that ketamine induces bursts of glutamate by obstructing the NMDAR on GABA interneurons [73]. GABAergic interneurons demonstrate heightened susceptibility to ketamine due to their tonic firing activity, which induces the removal of Mg^2+^ from the channel, thereby facilitating ketamine entry and subsequent blockade within the channel [63]. The glutamate bursts engender activity-dependent discharge of brain-derived neurotrophic factor (BDNF), which subsequently triggers downstream signaling cascades, including the Akt-mTORC1 pathway, heightening synapse density and function within the mPFC and increasing expression levels of synaptic protein (i.e., PSD95, GluA1, VGLUTs) [63].

Moreover, ketamine swiftly amplifies GABA function in the mPFC, evincing augmented gephyrin, VGAT, and GAD [63]. The up-regulation of GABA and glutamatergic neurotransmitter systems counteract the adverse effects of enduring stress exposure, precipitating an elevation in signal-to-noise ratio and fortifying signal integrity [63].

### 3.2. Neurotrophins

Neurotrophins, a group of growth factors, are crucially involved in the establishment, upkeep, and adaptability of neuronal networks [74]. Among the neurotrophin family members, BDNF is most abundant in both the periphery and the brain [75]. The “neurotrophic hypothesis of depression” posits that disrupted neurotrophic activity is associated with stress-induced depressive behavior and that antidepressant therapies promote the expression of BDNF [76]. BDNF has been implicated in neuroplasticity and is known to modulate synaptic connectivity, neuronal differentiation, outgrowth, and repair [77]. Abundant evidence demonstrates that changes in the expression of BDNF are seen in postmortem brain samples obtained from individuals diagnosed with depression [78, 79]. Additionally, there has been a consistent identification of reduced levels of BDNF in brain tissue samples from individuals exhibiting suicidality [80]. Furthermore, TrkB levels and the downstream signaling pathways of BDNF-TrkB, such as extracellular signal-regulated kinase (ERK) and Akt, appear to be reduced in suicide subjects [81]. In addition, Mitogen-activated protein kinase phosphatase 1 (MKP-1), a negative regulator of TrkB-ERK signaling, also increases in individuals with depression [81].

Chronic stress exposure has been associated with spine synapse dysfunction in the mPFC and hippocampus, potentially attributable at least in part to downregulated BDNF expression and reduced TrkB-mTORC1 signaling [82]. As mentioned above, ketamine-induced rapid glutamate bursts trigger the release of BDNF [63]. The upregulation of BDNF swiftly induces synapse dysfunction by stimulating TrkB-mTORC1 signaling and increasing the translation of synaptic proteins [83, 84]. Furthermore, BDNF-TrkB signaling is essential for ketamine-induced-antidepressant effects and synaptic potentiation in the hippocampus [85]. On the contrary, there is no evidence to indicate that conventional antidepressants can promptly trigger the release of BDNF [81]. Thus, BDNF is necessary for the rapid antidepressant actions of ketamine. However, additional investigations are warranted concerning the precise mechanisms underlying the BDNF-TrkB signaling cascade to determine if the interaction between antidepressants and the TrkB transmembrane domain constitutes a common mechanism across all antidepressant agents [84, 86].

### 3.3. Demyelination

Altered myelination is increasingly recognized as a key contributor to the etiology and therapeutic approach of depression, posing a significant challenge in establishing the primary causal factors [87]. Recent studies reveal disrupted integrity of white matter, abnormal structure of myelin, and impaired functioning of oligodendroglia in various cerebral regions implicated in depression, such as the dorsolateral PFC, the ACC, the hippocampus, and the corpus callosum [88, 89]. These findings align with postmortem analyses of brain tissue from depressive patients, which demonstrated decreased density of oligodendrocytes and abnormalities in the expression of genes related to oligodendrocytes [87]. Preclinical studies employing animal models have additionally reinforced the association between demyelination, dysfunction in oligodendrocytes, and behaviors characteristic of depression [90, 91].

Moreover, advancements in molecular investigations have yielded insights into dysregulated levels of messenger RNAs and proteins that exert pivotal functions in the differentiation and myelination of oligodendrocytes across distinct regions of the cerebral cortex [92]. Notably, within the context of depression, a remarkable 69% of significantly down-regulated genes were found to be involved in myelin, specifically myelin oligodendrocyte glycoprotein (Mog) and ermin (Ermn) [93]. However, investigations into the PFC of individuals with depressive disorder suggest that the primary changes in gene expression predominantly occur in oligodendrocyte progenitor cells (OPCs) and deep-layer excitatory neurons [94]. Within the OPC cluster, noteworthy alterations were detected in genes such as *prnf* (downregulated) and *kaze* (upregulated), indicating that *prnf* and *kaze* are involved in the pathogenesis of depression through altered myelination and synaptic plasticity [90, 94]. In addition, literature has documented diverse transcriptional modifications observed in male and female populations with depression, manifesting varied directions [95]. Notably, genes highly expressed in oligodendrocytes, namely MOBP and MAG, exhibited a notable decline in the ACC and dlPFC of female individuals suffering from depression [95]. In contrast, these genes displayed an upsurge in expression within the amygdala of women with depression [89, 95]. Strikingly, an inverse pattern of gene expression alterations emerged in males with depression, suggesting a multifaceted nature of the molecular mechanisms underpinning depression, which may possess sex-specific and region-specific disparities [89]. EphA4, a member of the ephrin receptor tyrosine kinases, stands out as a pivotal receptor on the neuronal post-synaptic membrane [96]. Its activation hinders the formation of myelin sheaths in the CNS and the peripheral nervous system [96, 97]. Mice subjected to stress-induced depression display downregulation of ubiquitination levels of brain proteins, leading to an increase in EphA4 protein levels, which result in demyelination and synaptic malfunctions [98]. Remarkably, discernible oligodendrocyte defects, diminished myelin thickness, and decreased myelin gene expression manifest in mice exposed to stress during adolescence, early life, and adulthood [96, 99]. Intriguingly, social reintegration in adulthood can redress these aberrations but fails to do so during early life, underscoring the presence of critical period-dependent oligodendrocyte maturation and myelin formation [100]. It is worth noting that the oligodendrocyte malfunctions may likely account for the individual differences in stress vulnerability [101].

The modulation of monoamine levels is also a vital pathway involved in stress-induced myelin loss [102-104]. The dopamine D2 receptor (D2R) is a prominent DA receptor subtype pivotal for oligodendrocyte development and function [104]. Furthermore, D2R activity is closely linked to anxiety-like and depression-like behaviors [104]. Notably, chronic stress triggers demyelination via activation of the Wnt/β-catenin signaling pathway and dopamine signaling mediated by D2R [105]. These factors contribute to defects in myelin formation and dysfunction in dopaminergic neuronal circuits, potentially leading to the etiopathogenesis of depression disorder [105]. During brain maturation, glutamate and GABA signaling are essential for activity-driven adaptive myelination, as they facilitate oligodendrocyte lineage cell development and function, mediated through multiple modes, including synaptic and extra-synaptic signaling [102]. It is worth noting that the OPCs situated within the CA1 region of the hippocampus receive direct synaptic inputs from neurons, encompassing both glutamatergic and GABAergic signaling pathways, impairing NMDAR-dependent LTP in pyramidal neurons of the S1 [101, 106]. In addition to disruptions in AMPAR membrane trafficking, compromised excitatory glutamatergic neurotransmission, and impaired extracellular glutamate uptake, these factors ultimately culminate in the manifestation of depressive-like behaviors in murine models [101]. Furthermore, oligodendrocyte development and myelin protein expression are also influenced by levels of 5-HT, and an elevated concentration of 5-HT in the brain is typically observed in cases of depression [107]. Previous investigations suggest that exposure to 5-HT reduces the number of myelinated internodes, changes the localization of paranodal contactin-associated protein, and alters the spatial organization without affecting cell death or oligodendrocyte density [103]. This diffused and enlarged pattern induced by 5-HT impacts axon-derived factors crucial for myelination [103]. Therefore, targeting interactions between neuronal-OPC synapses and elevated 5-HT levels may be key to suppressing oligodendrocyte proliferation and myelination for potential therapeutic interventions.

The neuromodulator functions of myelin and oligodendrocytes likely contribute significantly to dysfunctions in depression, including altered brain connectivity, heightened susceptibility to excitotoxicity, and decreased expression of endogenous antidepressant factors [87]. The genetic deletion of OPCs in adult mice induced depressive-like behaviors, which were ameliorated by repopulation of OPCs [101]. Hence, selective malfunctions of OPCs and oligodendrocytes have demonstrated causal precedence about the emergence of depressive-like symptoms in animal models, thus supporting the potential efficacy of oligodendrocyte-targeted therapeutics for depression and stress-related conditions. Recent studies indicate that ketamine ameliorates demyelination and facilitates remyelination, exerting long-lasting antidepressant effects [108, 109]. Specifically, ketamine promotes the differentiation of OPCs in mature oligodendrocytes by activating AMPAR signaling, which subsequently facilitates myelination [108].

### 3.4. Mitochondrial disruption

The "mitochondrial theory of depression" has garnered substantial support from a plethora of investigations elucidating the interconnectedness of depressive symptoms [1]. Furthermore, recent evidence reports that mitochondrial activity in the brain can predict stress-related behaviors in mice [110]. Disruptions within the intricate network governing mitochondrial dynamics, encompassing fusion, fission, and mitophagy, as well as impairments in mitochondrial structure and functionalities, such as diminished ATP synthesis, precipitate a series of aberrant alterations central to the debilitating pathological processes manifested in depression, thereby exacerbating outcomes [111, 112]. Noteworthy evidence from numerous studies substantiates the association between depression pathology and perturbations in metabolic pathways relevant to mitochondrial function within brain special regions implicated in emotional expression, attentional processes, and sensory perception, such as the PFC, insula, and basal ganglia [112, 113]. Furthermore, mitochondrial disruptions also engender the generation of free radicals, progressive impairments, increased apoptotic susceptibilities, and induction of inflammatory signaling cascades [114].

Recently, a correlation was discovered between depression and compromised mitochondrial function in dermal fibroblasts [5]. This pertains to various aspects of mitochondrial respiration, such as basal and maximal respiration, spare respiratory capacity, non-mitochondrial respiration, and ATP-dependent oxygen consumption, which exhibited lower values [5]. Additionally, depressive fibroblasts possessed diminished ATP levels and exhibited a hyperpolarized mitochondrial membrane potential [5]. The morphological alterations of mitochondria are contingent upon the metabolic phenotype of cells and are intricately regulated [115]. Peripheral fission of the mitochondrial outer membrane is facilitated by dynamic-related protein 1(Drp1), which interacts with different adapters. In contrast, central fission is propelled by the binding of Drp1 to the mitochondrial fission factor [115]. These distinctive forms of fission yield either defective mitochondria, which are subsequently eliminated via autophagy, or healthy mitochondria, which are utilized for mitochondrial proliferation [115]. Recent investigations have divulged that sustained exposure to social adversity induces neuronal mitochondrial fission through Drp1 mediation, which harms local ATP synthesis and impinges on neuronal AMPAR-dependent synaptic transmission, culminating in depressive-like behavior in murine models [116]. Therefore, Drp1 is a potent target for intervening in energy metabolism critical to stress-related depressive-like behavior [116].

Apart from producing ATP, mitochondria wield extensive regulatory power over redox homeostasis, cell apoptosis, and Ca^2+^ buffering, mainly regulating synapse development and synaptic plasticity [117]. For optimal function, mitochondria require dynamic equilibrium between fusion, fission, biogenesis, and mitophagy, a selective autophagic process targeting damaged mitochondria [118]. Recently, a study reported that mice subjected to chronic social defeats display damaged mitochondria in the basolateral amygdala (BLA) neurons and a reduction in mitochondrial DNA (mtDNA) copy number, indicating mitochondrial deficiency [118, 119]. The deficiency is not due to impaired mitochondria biogenesis, as shown by increased mtDNA replication and mtDNA mutation frequencies [118].

Further investigations suggest chronic social defeats incite mitochondrial impairments, activating the PINK1-Parkin mitophagy pathway in the amygdala, leading to a weakened BLA-BNST anxiolytic pathway and increased anxiety-like behaviors [119]. Additionally, SIRT1 activity, a well-established class III histone deacetylase, is implicated in the process of mitochondria mitophagy [120]. Sustained social defeats in mice upregulate SIRT1 expression in the NAc, and modulating SIRT1 activity via pharmacological or genetic methods regulates anxiety- and depression-like behaviors [121]. However, further investigation is essential to explore the potential link between SIRT1 activity modulation and anxiety- and depression-like behaviors mediated by mitochondrial mitophagy. In addition, the NAc is a crucial component of the reward circuitry and motivation systems in the brain, represents a pivotal hub in the pathophysiology of depression, and is intertwined with a distributed network of brain regions implicated in anxiety [57]. Consequently, highly anxious animals show reduced Mitofusin-2 (MFN2) levels, a mitochondrial outer membrane GTPase crucial for maintaining mitochondria-endoplasmic reticulum contacts [122]. Hence, regulating MFN2-mediated mitochondrial function and neuronal traits emerges as a key mechanism governing anxiety and motivated behaviors, providing a promising therapeutic target for managing anxiety and depression phenotypes [122].

In summary, there appears to be a close association between depression and mitochondrial dysfunction [113]. Nonetheless, the causative factors and the extent of mitochondrial dysfunction contribution to depression are yet to be comprehensively elucidated [113]. Ketamine's antidepressant response involves mitochondrial energy metabolism and antioxidant defense systems, activating glutamatergic neurotransmission and neuronal activity to induce LTP-like processes [123]. Mechanistically, ketamine activates mTORC1 to promote anabolic processes and AMP-activated protein Kinase to generate ATP, satisfying energy needs associated with synaptic potentiation [124]. Additionally, ketamine rapidly reduces ROS production, minimizing protein damage and achieving homeostatic redox regulation [124]. Thus, modulation of antioxidant mechanisms may be associated with the rapid antidepressant activity of ketamine.

### 3.5. Astrocyte dysfunction, energy metabolism disturbance, and inflammation

Astrocytes, the most populous cells in the brain, possess a heightened sensitivity to cerebral stressors, which induces alterations in their functional and structural characteristics [125]. Dysfunction of astrocytes contributes to aberrant resting-state functional connectivity observed in individuals with depression [126]. The mounting evidence indicates that the depressed brain is afflicted by a deterioration in the quantity, structure, and operation of astrocytes and that the impaired astrocytic purinergic system is likely implicated in the pathophysiology of depression [127, 128]. Through mechanisms reliant on calcium (Ca^2+^) dependent and independent processes, these cells detect synaptic activity and respond to neurotransmitters by releasing gliotransmitters [129, 130]. The actions of these gliotransmitters govern neuronal excitability and synaptic physiology, yielding significant impacts on brain function and behavior in animals [131]. Ketamine sustainably increases intracellular cAMP concentration (cAMPi) in astrocytes, even without activation of G-protein-coupled receptors [132]. The elevation in cAMP signaling inhibits stimulus-induced Ca^2+^ excitability, which may effectively attenuate vesicular channel delivery to the plasmalemma and alter the release or uptake of luminal cargo by modifying the fusion-pore structure [133].

**Figure 4.**
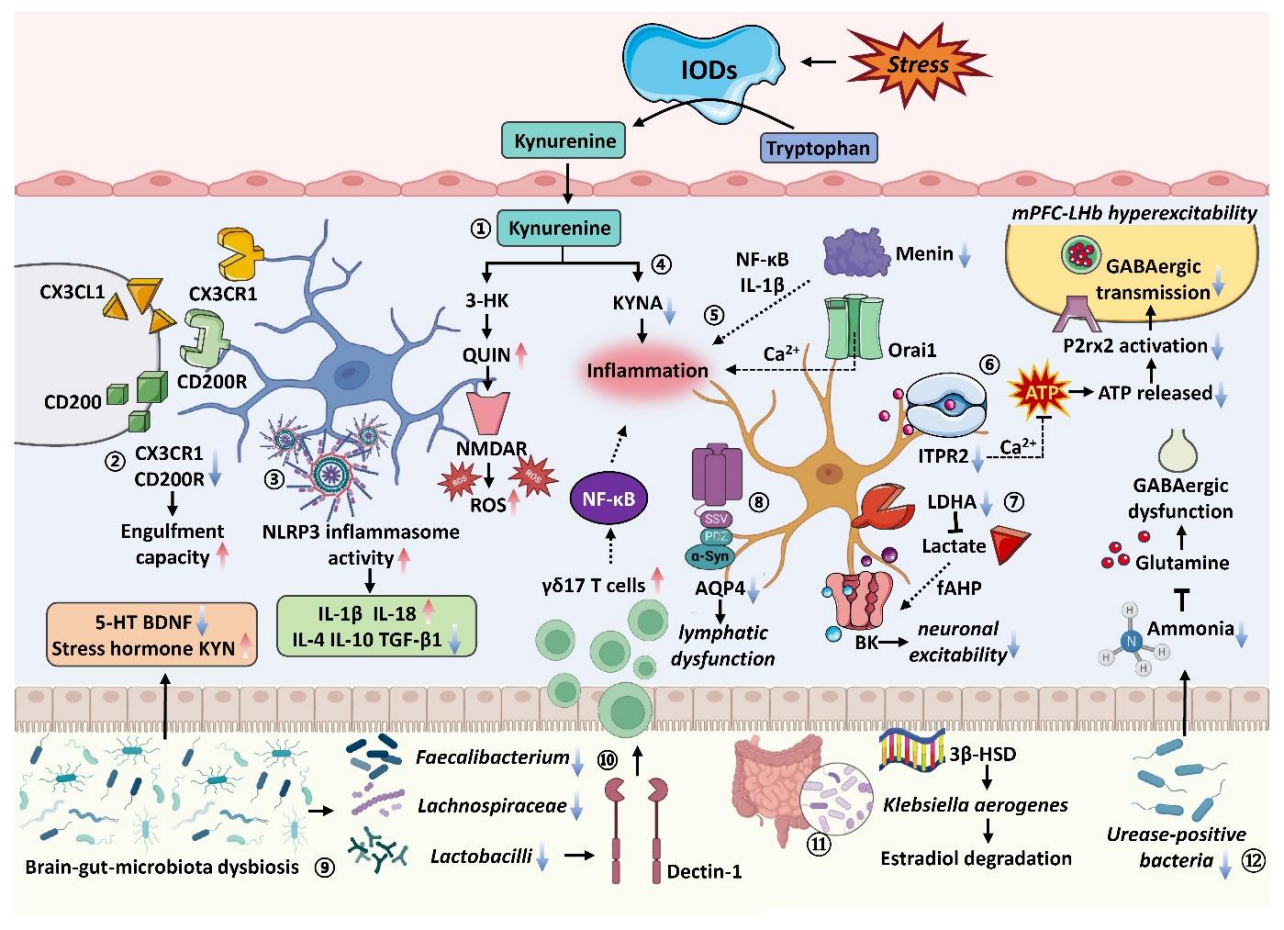
**The pathological mechanism of depression in microglia, astrocytes, and brain-gut axis**. (1) Microglial KYN pathway activation causes the generation of 3-HK and QUIN, potent neurotoxic characteristics; (2) disruption in CX3CR1 and CD200R increases microglial engulfment capacity; (3) the activation of the NLRP3 inflammasome elevates IL-1β and IL-18 and decreases IL-4, IL-10 and TGF-β1; (4) stress diminishes astrocyte-mediated KYNA release in KYN pathway, leading to inflammation response; (5) in addition, the ablation of menin-induced abnormal activation of NF-κB and IL-1β production and Orai1 channels dysfunction also drive neurotoxic inflammation; (6) damage to ITPR2-dependent ATP release leads to decreased P2rx2 activation and ultimately results in aberrant hyperexcitability of the mPFC-LHb pathway; (7) deficiency in LDHA within astrocytes diminishes L-lactate production, consequently impairing neuronal excitability by augmenting the activity of BK channel-mediated fAHP; (8) aberrant glutamate reduced AQP4 expression, impairing astrocyte-mediated lymphatic transport; (9) Stress disrupts the composition of the microbiota, leading to the decline of 5-HT and BDNF expression, elevation of plasma stress hormone levels and KYN; (10) gut microbiota dysbiosis and decreased levels of lactobacilli increase γδ17 T cells expression by dectin-1 signaling, facilitating neuroinflammation; (11) heterologous expression of 3β-HSD led to the degradation of estradiol in premenopausal females with depression; (12) ammonia reduction associated with decreased urease-positive bacteria, restricting the availability of cerebral glutamine and inducing GABAergic dysfunction.

Recent investigations have unveiled the potential therapeutic implications of astrocytic Ca^2+^ signaling in maintaining brain circuitry and managing depressive-like conditions [130]. Specifically, the 5-HT triggers the release of glutamate from astrocytes via 5-HT2 receptor activation, which in turn activates neuronal mGluR1 and NMDAR, driving synaptic plasticity by 5-HT in the mPFC [130]. Conversely, mice presenting depressive-like behavior exhibit diminished astrocytic Ca^2+^ signaling mediated by 5-HT, impairing the release of glutamate and leading to aberrant synaptic deficits associated with 5-HT dysfunction [130]. Notably, elevated levels of glutamate can induce astrocytic pyroptosis, thereby impairing astrocyte-mediated lymphatic transport [134]. However, ketamine can reverse lymphatic dysfunction by suppressing the expression of pyroptosis-related proteins Nlrp3/Caspase-1/Gsdmd-N [134]. In rodent depression models, a mechanism of astrocyte-neuron communication in the lateral habenula (LHb) involving the astrocytic potassium channel Kir4.1 has been observed to elicit neuronal bursts. [135, 136]. The membrane hyperpolarization extent and bursting activity level of LHb neurons are tightly regulated by the expression level of Kir4.1 on astrocytes [135]. Furthermore, under depressive conditions, Kir4.1 upregulation may cause a decrease in extracellular potassium (K^+^) and hyperpolarization of LHb neurons [135]. In ketamine-treated astrocytes, the trafficking rate of inwardly rectifying potassium channel (Kir4.1) vesicles is reduced, decreasing the surface density of Kir4.1 and attenuating depressive symptoms in rats [137]. Interestingly, ketamine may induce changes in synaptic plasticity by modulating extracellular K^+^ homeostasis, resulting in sustained enhancement of excitatory synapses required for its antidepressant effects [137]. Furthermore, the hyperpolarization of neurons may result in the de-inactivation of T-type voltage-sensitive calcium channels, subsequently leading to NMDAR-dependent bursts that hat enhance the inhibition of downstream monoaminergic centers, resulting in an intensified output of LHb and exacerbating depression [135, 136]. However, ketamine inhibits the NMDAR-dependent burst activity of LHb neurons, thereby alleviating the suppression of monoaminergic reward centers and swiftly ameliorating mood.

ATP is an essential glial transmitter for regulating depressive-like behaviors, regulated by the purinergic P2X2 receptor (P2rx2), which regulates inhibitory synaptic transmission [127, 138]. Previous studies have shown that low ATP abundance in the brain, especially the PFC and hippocampus, is vulnerable to chronic social defeat [127, 139]. Inositol 1,4,5-trisphosphate receptor type 2 (ITPR2), a crucial regulator of calcium ion transmembrane transport activity, mediates the release of ATP from astrocytes through Ca^2+^-dependent exocytosis [140]. Further studies show that knockout of ITPR2 results in reduced ATP release from astrocytes and a depressive phenotype in mice [141]. As mentioned above, activating the mPFC-LHb pathway can induce depressive-like behavior [130]. Interestingly, ITPR2 knockout mice also exhibit reduced neuronal inhibitory post-synaptic currents in the mPFC-LHb pathway [141]. Therefore, in chronic stress, damage to ITPR2-dependent ATP release leads to decreased extracellular ATP levels, reducing P2rx2 activation in inhibitory neurons and ultimately resulting in aberrant hyperexcitability of the mPFC-LHb pathway [141, 142]. In addition, the decrease in ATP release from astrocytes is also associated with the downregulation of GR expression in the mPFC following chronic stress, mediated by the PI3K-AKT signaling pathway [143].

Multiple evidence consistently highlights the pivotal role of disrupted glucose metabolism in the pathogenesis of depression [144, 145]. Particularly noteworthy, the human PFC is recognized to exhibit a heightened glycolytic state during rest and demonstrably amplifies its glycolytic capacity upon cerebral activation [146, 147]. Numerous investigations have implicated the exportation of glycolysis-derived L-lactate from astrocytes to neurons as a determinant influencing their survival and activity, thereby suggesting a pivotal regulatory role of glycolysis and the lactate shuttle in governing brain physiology [148, 149]. Notedly, astrocytic lactate dehydrogenase A (LDHA) modulates neuronal excitability and depressive-like behaviors by maintaining lactate homeostasis, particularly within the PFC. [146]. Subsequent exploration revealed that deficiency in LDHA within astrocytes diminishes L-lactate production, consequently impairing neuronal excitability by augmenting the activity of the large-conductance Ca^2+^-activated potassium channel-mediated fast afterhyperpolarization, ultimately culminating in the manifestation of depression-like phenotypes [146].

Astrocytes drive brain inflammation by releasing inflammatory and chemotactic factors, contributing to the development of depression. Inhibiting astrocyte activation improves depressive behaviors such as anhedonia and despair [150, 151]. Reduced levels of multiple endocrine neoplasia type 1 (Protein: Menin; Gene: Men1) in astrocytes lead to a depressive-like phenotype, including depressed mood and impaired social interaction [150]. Similarly, in human *Men1*, the discovery of SNP rs375804228 is linked to an increased risk of depression and results in abnormal activation of NF-κB and IL-1β production [150]. Mechanically, the ablation of menin decreased the capacity for transcriptional repression within the pvalb promoter region, culminating in a pronounced upregulation of PV expression levels and resulting in a depressive-like phenotype in mice [152]. Recent studies show that ketamine stabilizes menin levels by inhibiting the kinase activity of protein kinase A, thereby maintaining its antidepressant effects [152]. As previously mentioned, as a non-excitable cell, astrocyte activity mainly depends on intracellular calcium elevation [153]. Aberrant calcium signaling in astrocytes also mediates neurotoxic inflammation [154]. Store-operated calcium entry, one of the pathways for non-excitable intracellular calcium, is mediated by stromal interaction molecule 1 and the calcium-release-activated calcium channel protein Orai1-3 [155]. Recent studies show that Orai1 channels are essential for regulating reactive astrocyte proliferation, driving inflammation-induced astrocytic Ca^2+^ signaling, and synthesizing and releasing various pro-inflammatory mediators [153].

### 3.6. Microglia dysfunction and inflammation

The characteristic structure and function of microglia in developing and adult brains are linked to the pathogenesis of depression (Fig. 4) [156]. Researchers conducted a cross-sectional assessment to explore the correlation between microglial activation and neural inhibition using TSPO-V_T_ in depressed patients and non-depressed controls [157]. Additionally, hey also investigated the association of this neuroinflammatory marker with untreated major depression duration, total illness duration, and antidepressant exposure [157]. Indeed, Microglial activation levels (TSPO-VT) were notably linked to persistent severe depression, particularly in the ACC, frontal cortex, and insula of patients without long-term antidepressant [157]. Although the data is cross-sectional, evidence suggests that antidepressant treatment alleviates the progressive increase in TSPO-V_T_ within the duration of untreated depression, indicating a relationship between accumulated disease burden and microglia activation [157, 158]. Correspondingly, other studies have obtained similar results [159, 160].

The bidirectional communication between microglia and neurons occurs through soluble mediators such as neurotransmitters, chemokines, and cytokines [161]. CX3CL1 (fractalkine) and CD200 (OX-2 membrane glycoprotein) are noteworthy amongst these molecules due to their specific localization on distinct cell types. Neurons predominantly express CX3CL1, whereas microglia express its receptor, CX3CR1 [161]. Similarly, neurons express CD200, while microglia possess CD200R [161]. The interaction of CX3CL1 and CX3CR1 supports the maintenance of functional stability by microglia [162, 163]. Prior studies indicate that disrupted CX3CR1 signaling results in social withdrawal, reduced behavioral adaptability to social dominance, and increased repetitive behavior [163]. Furthermore, Cx3cr1-deficient mice displayed a reduction of microglia during the early postnatal period, followed by subsequent deficits in synaptic pruning [162]. Dysfunctional synaptic pruning has been associated with compromised synaptic transmission, impaired neural circuit formation, diminished social interaction, and heightened repetitive behavior, all of which are observed in numerous neuropsychiatric disorders [162]. Hence, microglia-mediated disruption of synaptic pruning may contribute to the pathogenesis of neurodevelopmental and neuropsychiatric conditions [162]. Furthermore, another investigation proposed that early-life inflammation induces enduring maladaptation of ACC glutamatergic neuronal spines in response to stress [164]. This, in turn, disrupts Cx3cr1-mediated microglial engulfment capacity, ultimately facilitating the emergence of depression-like symptoms during adolescence [164]. The majority of the data concerning the involvement of the CD200-CD200R pathway in the pathogenesis of depression has been gleaned from investigations utilizing diverse stress-inducing protocols in animal models [161]. Animal models for depression subjected to early-life social isolation showed a significant reduction in the expression of the CD200R in the hippocampus, which is essential for promoting microglial quiescence [165]. Furthermore, acute and chronic stress-induced changes in CD200-CD200R signaling in corticolimbic circuitry [166]. Intriguingly, exposure to inescapable tail shock has been shown to decrease CD200R levels in the hippocampus and the amygdala's basolateral and central nucleus [167]. However, a paradoxical finding has been reported, wherein unavoidable foot shocks were observed to decrease the transcriptional activity of CD200R in the hypothalamus but not in the hippocampus [167]. These apparent inconsistencies may be related to the employment of different stress exposure paradigms, warranting continued investigation.

It has been reported that depression augments the permeability of the blood-brain barrier [168], allowing for peripheral cytokines to infiltrate the CNS and trigger an inflammatory response in microglia, resulting in an aberrantly inflamed pro-inflammatory cascade [169, 170]. Among the various cytokines implicated in the communication between immune cells of the peripheral system and the brain, TNF-α, IL-1β, IL-18, and IL-6 have been identified as crucial players, with elevated levels of these mediators associated with depression [171-173]. In addition, the activation of the NLRP3 inflammasome has been detected in both individuals and animal models suffering from depression [174]. Previous investigations have revealed the activation of the NLRP3 inflammasome in blood cells obtained from patients suffering from depression [174]. Moreover, the serum levels of IL-1β and IL-18 were elevated, implying that the NLRP3 inflammasome potentially exerts a pivotal function in orchestrating the progression of depressive symptoms [174]. Conversely, dysregulation of anti-inflammatory cytokines might also contribute to susceptibility to stress and microglia-mediated symptoms of depression.

Reduced levels of cytokines such as IL-10, IL-4, and TGF-β1 have been observed in both the plasma of patients with depression and the brain tissue of animal models of depression [175, 176]. Evidence from studies involving microglial-specific knockouts of IL-10 has demonstrated induction of depressive-like behavior in mice, along with elevated IL-6/IL-10 ratios, indicating an imbalanced pro- and anti-inflammatory cytokine milieu may promote inflammation [177]. Moreover, deficiency in IL-4 compromises stress resilience, while increasing cytokine levels attenuates depressive symptoms [168, 178]. IL-4 has been shown to facilitate the reprogramming of microglia towards an arginase 1 (Arg1) phenotype, which is essential for maintaining optimal neuroprotective and regenerative functions, thereby promoting brain homeostasis [179]. Additionally, recent research has highlighted the role of hippocampal IL4-induced Arg1+ microglia in promoting neurogenesis, which helps protect against depressive-like symptoms in the context of exposure to chronic mild stress [180].

Following repeated administration of ketamine, multiple peripheral inflammatory cytokine levels were downregulated, and these changes exhibited a significant correlation with the amelioration of depressive symptoms [181]. Moreover, the sustained antidepressant actions of ketamine are related to microglial ERK-NRBP1-CREB-BDNF signaling [182]. Additionally, both peripheral blood of depressive patients and depressive-like mouse models show significantly decreased levels of circular RNA DYM (circDYM) [183]. The reinstatement of circDYM expression notably mitigates depressive-like behavior and suppresses microglial activation [183]. Particularly, circDYM functions as an endogenous microRNA-9 (miR-9) sponge [183]. Further investigation reveals that overexpression of circDYM in the hippocampus suppresses the activity of miR-9, ultimately reducing microglial activation and alleviating depressive-like behavior [183]. Currently, extracellular vesicle-mediated delivery of circDYM has been discovered to suppress microglial activation, attenuate blood-brain barrier leakage substantially, reduce peripheral immune cell infiltration, and ameliorate astrocyte dysfunction induced by stress, effectively alleviating chronic stress-induced depressive-like behavior [183]. Therefore, further investigation is warranted to determine whether ketamine can restore circDYM expression.

### 3.7. Kynurenine pathway

The kynurenine (KYN) pathway, a cascade of enzymatic processes governing the metabolism of the indispensable TRP, has been subject to considerable investigation in the context of inflammation and the emergence of depressive-like symptoms [184]. Recent studies have notably illuminated the intricate interconnectedness between the KP and numerous pathways underlying depression, including inflammation, immune cell activity, as well as acute, chronic mild, and early-life stress [185, 186]. Moreover, the downstream metabolites generated within the KYN pathway exhibit variability contingent upon the cell type engaged [187]. Astrocyte-mediated KYN pathway activation gives rise to kynurenic acid (KYNA), which exhibits neuroprotective properties through its capacity to counteract glutamate overflow [187]. In contrast, microglial KYN pathway activation may result in the generation of either 3-hydroxykynurenine (3-HK) and quinolinic acid (QUIN) or anthranilic acid [187]. Of note, QUIN exhibits robust neurotoxic properties through the facilitation of NMDAR activation, leading to the generation of reactive oxygen species (ROS) and exacerbation of inflammation via the upregulation of chemotactic molecules [188].

The enzyme indoleamine 2,3-dioxygenase (IDOs) catalyzes the conversion of TRP to KYN in the initial step of the KYN pathway [189]. Recent investigations have posited that IDOs serve as pivotal molecular mediators in the manifestation of depressive-like behavior induced by inflammation, potentially through augmenting the conversion of TRP to KYN [190]. Subsequently, KYN crosses the blood-brain barrier and ultimately gives rise to excessive QUIN and diminished KYNA in the brain [189]. Nevertheless, the multifaceted implications arising from metabolic processes along different branches of the KYN pathway, as well as the clinical significance of the resultant end products, are still not fully understood. [191]. Clinical studies conducted on depressed individuals have produced inconsistent outcomes attributable to limited sample sizes, as well as variations in symptom severity and sampling methods (blood, cerebrospinal fluid, or urine) [191]. KYN pathway may be involved in the antidepressant mechanism of ketamine [192]. Previous studies have suggested that ketamine administration reduced levels of QUIN, IDO, and the KYN/ TRP ratio [192, 193]. Additionally, modest associations were observed between early alterations in serum KYNA levels and the KYNA/KYN ratio, approximately 24 hours post-initial ketamine infusion, and the subsequent antidepressant effects in patients with depression [193]. Consequently, the KYN pathway manifests as a potential target of therapeutic intervention for the antidepressant effects of ketamine.

### 3.8. Hypothalamic-pituitary-adrenal (HPA) Axis dysfunction and Stress Exposure

Regarded as the most extensively researched and established risk factor for depression, stress exposure, especially during early life, has been associated with numerous symptoms of depression [194]. Multiple studies have documented structural and functional changes in neuronal activity after exposure to chronic stress [194, 195]. Abundant investigations have delineated intricate molecular cascades from activating the hypothalamic-pituitary-adrenal (HPA) axis stress response in neuronal populations [196]. Specifically, Vasopressin (AVP) and corticotropin-releasing factor (CRF) originating from the hypothalamus regulate the activity of the HPA axis [197]. ACTH is released by the pituitary corticotrope when AVP and CRF are activated [197]. Glucocorticoids discharged from the adrenal cortex engage with receptors across diverse target tissues, encompassing the HPA axis, inhibiting the secretion of CRF, ACTH, and AVP [197].

Cortisol released by the adrenal glands binds to mineralocorticoid receptors (MR) in the brain with high affinity, but its affinity for glucocorticoid receptors (GR) is lower [198]. GR is widely distributed throughout the primate brain, while MR is primarily localized in the hippocampus [198]. The powerful effects of cortisol mainly occur through hippocampal MR, whereas GR mediates feedback effects at the pituitary and activated brain regions like the amygdala [199]. Dysregulation of MR and/or GR within the HPA system is speculated to underlie severe depression [200]. The processing of emotion and cognitive functioning in the brain can be impacted by increased glucocorticoid levels, with specific alterations observed in the mPFC [201], the hippocampus [202], and the amygdala [203]. The mPFC is responsible for executive functions and emotional processing, the hippocampus for cognitive function, and the amygdala for emotion processing [204]. Moreover, cortisol disrupts functional connections in regions related to emotion processing and adaptation. Chronic stress weakens connections from the basolateral amygdala to the mPFC, leading to increased amygdala excitability and impaired cognitive processing [205]. Over 40-60% of depression patients exhibit high cortisol secretion, which has a genetic component [206]. The genes NR3C1 (GR) and NR3C2 (MR), associated with cognitive performance, are predictive of cortisol dysregulation [200]. Furthermore, higher cortisol levels are negatively correlated with cognitive performance, with GR genetic variation linked to attention and working memory and MR implicated in verbal memory [200]. In addition, GR target gene FKBP5 is implicated in depression, as its polymorphisms are associated with HPA axis parameters, response to antidepressant treatment, and recurrence of depressive episodes [207, 208]. Moreover, through protein-protein interactions, FKBP51 influences the signaling of other depression-relevant pathways, such as GSK3β, BDNF, and nuclear factor kappa B, thereby connecting it to inflammation, the immune system, and autophagy [46].

Nonetheless, a wealth of preclinical investigations has substantiated the pivotal involvement of non-neuronal cellular constituents in orchestrating the adaptive responses to sustained stress-induced perturbations of homeostasis [209]. Recent research has revealed that early-life stress increases the production of endogenous glucocorticoids, which triggers the expression of the GR in astrocytes, driving the engulfment receptor Mertk and significantly increasing the number of LAMP2+ lysosomes in various cortical regions [210]. Similarly, MR expression in astrocytes confines the activation of XBP1, a transcription factor that fosters pro-inflammatory signaling via the upregulation of cytokine expression in astrocytes, inducing behavioral deficits resulting from chronic stress [211]. Furthermore, early-life stress also causes an increase in synaptic pruning in cortical layers 2, 3, and 5 of the primary somatosensory cortex (S1) and a decrease in excitatory synaptic density, leading to modified patterns of neural activity and heightened helplessness behavior [210]. Furthermore, synaptic pruning occurring in the primary S1 is a crucial driving force steering stress-induced behavioral impairment [210]. Notably, S1 presents a critical hub in neurodevelopment, necessitating further exploration of its connectivity to other cerebral regions implicated in emotional behavior [210, 212].

Numerous pharmacological agents targeting the neuroendocrine stress axis have been tested as potential therapeutics for depression [213]. Given the heterogeneity of patients regarding HPA axis dysfunction, it may be imperative to undertake genetic or functional evaluations at baseline to discern individuals who will respond favorably [213]. Remarkably, ketamine treatment has exhibited promising outcomes in individuals manifesting psychotic depression [214]. A single administration of ketamine ameliorated depressive-like behaviors, reduced circulating cortisol (CORT) levels, and rescued both the expression and nuclear translocation of GR [214]. Consequently, altered CORT concentrations after stress may serve as a potential predictive measure for susceptibility to depression in clinical settings [214]. Moreover, emerging data propose that the temporal administration of ketamine might influence HPA axis activity, given the classic observation that the circadian release of glucocorticoids aligns with diurnal rhythms [215]. Interestingly, there is heightened HPA axis activity in response to ketamine administration during inactivity [215].

### 3.9. Brain-gut-microbiota axis (dysbiosis)

The gut microbiota hypothesis suggests a pivotal role for the gut microbiota in depression pathology through the gut-brain axis [216]. Clinical investigations have revealed significant differences in gut microbiota composition between individuals with depression and healthy controls [216]. Specifically, individuals with depression exhibit marked alterations in the relative abundance of Firmicutes, Actinobacteria, and Bacteroidetes [217]. Currently, the consistent evidence indicates a perturbation in the microbial composition within the context of depression, including a reduction in the abundance of specific taxa responsible for butyrate synthesis, namely *Faecalibacterium*, and Lachnospiraceae, alongside the diminished short-chain fatty acid (SCFA)-producing lactobacilli [218]. In addition, the 3β-hydroxysteroid dehydrogenase (3β-HSD) gene was successfully identified as the genetic determinant responsible for encoding the estradiol-degrading enzyme in *Klebsiella aerogenes*, and heterologous expression of 3β-HSD led to the degradation of estradiol in premenopausal females with depression [219]. Interestingly, gut microbiota expression of 3β-HSD may be linked to depressive symptoms via testosterone degradation [220]. Similarly, recent research demonstrated that *Morganella* has been suspected of a potential causal effect on depression [221]. The transplantation of fecal microbiota can induce the transmission of depressive symptoms [217]. Fecal microbiota transplantation experiments in germ-free mice observed that colonization with “depression microbiota” from depressive patients induced depression-like behaviors compared to “healthy microbiota” from healthy individuals [217]. These mice primarily exhibited disruptions in microbial genes and host metabolites related to carbohydrate and amino acid metabolism, suggesting specific microbiota phenotypes induce depressive symptoms via metabolic alterations [217]. Subsequent investigations indicated that depressive symptoms are able to transfer between subjects, further supporting the regulatory role of gut microbiota in psychological states [222].

Stress disrupts the composition of the microbiota, leading to various detrimental effects and significantly increase the susceptibility to depression [223, 224]. The prominent changes observed in stressed mice include a noteworthy decrease in the Lactobacillus population and an elevated level of circulating KYN [225]. It has been reported that the administration of Lactobacillus effectively ameliorates cognitive dysfunction and biochemical abnormalities induced by chronic restraint stress [224, 225]. The potential mechanisms underlying this protective effect of Lactobacillus involve the direct inhibiting IDO1 expression and subsequent decreasing KYN levels [225]. In addition, chronic stress leads to dysbiosis of the gut microbiota and decreased levels of lactobacilli, which promotes an increase in interleukin (IL)-17-producing γδ T cells (γδ17 T cells) in the intestinal tract mediated by dectin-1 signaling [226]. These cells migrate to the meninges and activate NFκB, thereby facilitating the occurrence of neuroinflammation and depression-related behaviors, revealing the regulation of brain function by gut immune cells [226]. Furthermore, the connection between intestinal ammonia and host stress vulnerability is established by maintaining cerebral glutamine availability [227]. Chronic stress significantly reduces blood, colon, fecal, and cerebrospinal fluid ammonia levels in mice, which is associated with decreased urease-positive bacteria in the intestinal tract [227, 228]. Upon entering brain tissue, ammonia is rapidly taken up by astrocytes and converted into glutamine-by-glutamine synthetase [229]. The released glutamine from astrocytes is selectively transported to GABAergic neurons through specific glutamine transporters, facilitating GABA synthesis [230]. Importantly, abnormally low blood ammonia levels restrict the availability of cerebral glutamine, hindering synaptic replenishment of pre-synaptic GABA in GABAergic neurons and contributing to vulnerability to stress-induced cortical GABAergic dysfunction [230]. In summary, chronic stress disrupts the balance of urease-positive bacteria in the intestinal tract, resulting in decreased endogenous ammonia levels, and leads to impaired glutamine synthesis in the mPFC and compromised GABAergic synaptic transmission, ultimately contributing to the development of depression [227].

Gut microbiota dysbiosis emerges as a pivotal determinant in depression pathogenesis [231, 232]. Although gut microbiota profiles in depression vary across studies, the consistent revelation of significant alterations underscores the potential of gut microbiota as an innovative therapeutic target for depression [231, 232]. Recent evidence suggests that the antidepressant effects of ketamine may be correlated with the normalization of dysregulated gut microbiota [233]. For example, the phylum *Actinobacteria* and the class *Coriobacteriia* may serve as potential biomarkers for the antidepressant efficacy of ketamine [233]. Notably, ketamine increases probiotic genera while decreasing pathogenic genera [234, 235]. Significant improvements were observed in the levels of *Mollicutes*, *Butyricimonas*, *Bacteroidales*, and *Clostridiales*, while *Ruminococcaceae*, *Clostridium*, and *Deltaproteobacteria* showed marked reduction [234, 235]. Furthermore, findings from other research supported that ketamine increases low-abundance bacterial genera, including *Lactobacillus*, *Turicibacter*, and *Sarcina*) and decreases opportunistic pathogens, including *Ruminococcus* and *Mucispirallum* [236]. Taken together, diverse alterations in colonic microbiota may contribute to the sustained antidepressant and anti-inflammatory effects of ketamine [234-236]. To fully grasp the relationship between gut microbiota and ketamine's antidepressant effect, thorough investigations at the bacterial species level are essential. Variations in bacterial species within the same genera can have distinct or opposing effects on depressive symptoms.

### 3.10. Other interrelated pathways

*PH homeostasis.* The Na+/H+ exchanger 1 (NHE1) plays a key role in regulating intracellular (pHi) and extracellular pH (pHe), influencing various physiological and pathological processes [237]. Recent studies suggest a potential involvement of NHE1 in neuroplasticity deficits and the development of depressive behaviors [237]. Exposure to stress instigates synaptic plasticity alterations linked to depression pathogenesis by hippocampal NHE1 deficits [238]. Specifically, the deficits in NHE1 are attributed to its ubiquitination and degradation by activating E3 ubiquitin ligase cullin4A, ultimately resulting in intracellular acidification [238]. Hence, targeted regulation of NHE1 expression or functionality offers a novel therapeutic approach to enhance the efficacy of existing antidepressant interventions by maintaining pHi homeostasis [238].

**Figure 5.**
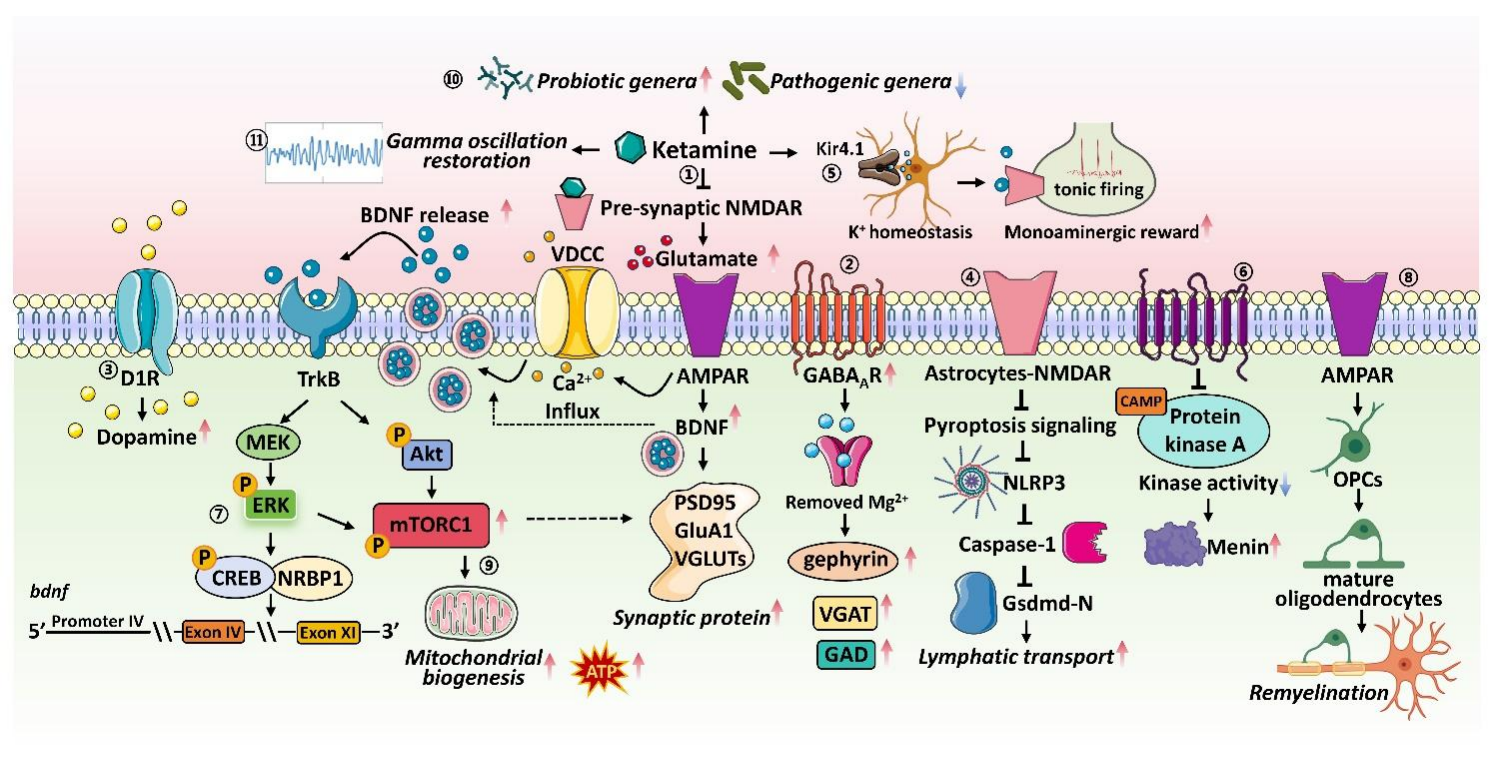
**Summary of the rapid antidepressant action of Ketamine in depression**. (1) ketamine induces bursts of glutamate, leading to increase in BDNF, alongside increased expression of synaptic protein levels (i.e., PSD95, GluA1, VGLUTs); (2) Moreover, ketamine swiftly amplifies GABA function augmenting levels of VGAT, GAD, and gephyrin; (3) ketamine broadly impacts the dopaminergic regulatory system, resulting in increased firing of dopamine neurons and enhanced dopamine release; (4) ketamine reverse lymphatic dysfunction by suppressing the expression of pyroptosis-related proteins Nlrp3/Caspase-1/Gsdmd-N; (5) ketamine modulates extracellular K^+^ homeostasis by reducing astrocytes-Kir4.1, relieving inhibition on downstream monoaminergic reward centers; (6) ketamine stabilizes menin levels by inhibiting the kinase activity of protein kinase A, thereby maintaining its antidepressant effect; (7) in addition, the sustained antidepressant actions of ketamine are related to microglial ERK-NRBP1-CREB-BDNF signaling; (8) ketamine promotes the differentiation of OPCs in mature oligodendrocytes through activation of AMPAR signaling, facilitating remyelination; (9) ketamine treatment activates mTORC1 to promote anabolic processes including protein synthesis and mitochondrial biogenesis; (10) in brain-gut-microbiota axis, ketamine appears to increase probiotic genera while decreasing pathogenic genera; (11) ketamine treatment ameliorates depression-like behaviors in animal models with impairing edge gamma oscillations.

*Antiviral immune response.* In the context of viral infection, antiviral innate immunity serves as the frontline defense mechanism against viral pathogens [239]. Upon viral infection, innate immune cells activate pattern recognition receptors to detect specific pathogen-associated molecular patterns of the viruses [239, 240]. Subsequently, this recognition triggers the production of IFN-I, which in turn activate a cascade of intracellular signaling events through the phosphorylation of tyrosine kinase 2 (Tyk2), facilitating the expression of interferon-stimulated genes (ISGs) and bolstering the innate antiviral capacity [240]. As mentioned above, alterations in levels of AVP in the HPA system are present in depression [197]. Intriguingly, recent investigations indicate that abnormally heightened AVP levels in depressive individuals lead to a decline in macrophage AHI1 expression, provoking a significant dampening of ISG expression and diminishing the efficiency of interferon-mediated antiviral innate immunity [241]. Additionally, AHI1 is critical for maintaining the stability of IFN-I signal transduction [241]. Therefore, the reduction of AHI1 ultimately results in the downregulation of Tyk2 and subsequent attenuation of IFN-I signaling activity in macrophages obtained from depression [240]. It is worth noting that individuals with depression are more susceptible to viral infections, and viral infections can contribute to the development of depression [241]. Currently, only the analgesic drug meptazinol has been discovered to upregulate AHI1 and Tyk2 expression in a mouse model of depression, suppressing viral infection [240]. However, the intricate mechanisms underlying the impact of depression on antiviral innate immunity have yet to be thoroughly investigated, necessitating further research endeavors to provide comprehensive insights into this domain.

*Gamma oscillations.* Coherent gamma oscillations (30-80 Hz) establish close relationships among brain regions, facilitating the transmission of information by enhancing neuronal excitability [242]. Depressed individuals display notable disruptions in functional network connectivity [242, 243]. Emerging evidence suggests the olfactory bulb is a potential source of gamma oscillations, projecting to brain regions including the piriform cortex (PirC), olfactory bulbectomy, NAc, and amygdala, contributing to gamma oscillatory network activity [244]. The suppression of olfactory bulb neurons or OB-PirC pathway results in diminished gamma oscillation power, triggering depression-like behaviors in rodents, including reduced hedonic response, impeded environmental adaptation, and heightened immobility in response to stress [244]. Conversely, ketamine treatment ameliorates depression-like behaviors in animal models with impaired edge gamma oscillations, underscoring the potential of gamma oscillation restoration as a therapeutic approach for depressive symptoms [244] (Fig. 5).

## 4. Co-morbid other diseases and depression

### 4.1. Stroke

PSD is the most common neuropsychiatric comorbidity of stroke [86]. Notably, many stroke patients develop depression, leading to more significant disability and increased mortality rates [86]. The precise pathophysiology of PSD remains unclear, involving mechanisms such as glutamatergic systems, monoaminergic, gut-brain axis dysfunction, and HLA [245]. In addition, neuroinflammation also plays a role in PSD pathogenesis, involving glial cells inducing the release of cytokines (TNF-α, IL-1, IL-6) and monocyte-derived macrophages [246]. For example, the downregulation of miR34b-3p in hippocampal neurons after stroke leads to increased expression of eIF4E, activating microglia-mediated neuroinflammation and ultimately inducing PSD [247]. Interestingly, the antidepressant effect of ketamine is mediated by eIF4E in cell-specific translation [30]. Additionally, ketamine administration in rodents after middle cerebral artery occlusion effectively improved ischemic brain injury and behavioral deficits [248]. Further research found that ketamine treatment rapidly demonstrates significant and persistent antidepressant-like effects in depressive stroke models by regulating NMDAR/CaMKII-mediated synaptic plasticity [7]. Hence, ketamine may contribute to ameliorating behavioral abnormalities after stroke, warranting further investigation.

### 4.2. Posttraumatic stress disorder

PTSD is a severe mental health disorder characterized by intensified fear responses persisting for months or even years after intensely aversive events [249]. Furthermore, prolonged fear behavior makes individuals with PTSD more susceptible to psychiatric illnesses, leading to a high incidence of suicide [250]. Recent findings suggest that the higher-order thalamus, including the thalamic reticular nucleus-posteromedial complex and frontal association cortex, mediates excessive defensive behaviors in PTSD [251]. Further reports suggest that hyperactivity in the mediodorsal thalamic nucleus preferentially activates downstream PV+ interneurons, disrupting the local excitatory-inhibitory balance in the prefrontal cortical microcircuitry, thereby delaying fear memory extinction [249].

PTSD and depression share a common feature of dysregulation in the HPA axis following exposure to traumatic events, manifested as increased GR sensitivity and enhanced negative feedback inhibition of the HPA axis [252]. FK-506 binding protein 5 (FKBP5) plays an indispensable role in the signaling cascade of glucocorticoid receptors [253]. Notably, the FKBP5 gene is a pivotal regulatory gene for the FKBP5 protein and has been explored for a potential linkage with depression and post-traumatic stress disorder co-occurrence [253]. Glutamatergic synaptic dysfunction is implicated in the neurobiology of depression, with PTSD patients exacerbating psychopathology through disruption of glutamatergic synaptic strength in the PFC [254]. Similarly, the presence of synaptic downregulation in depression and PTSD [255]. For instance, a decline in synaptic density among depressive patients with comorbid PTSD is indicated by the detection of a radioligand targeting synaptic vesicle protein 2A [256]. A large-scale clinical trial has shown that ketamine mitigates the negative impact of traumatic memories in PTSD by interfering with memory reconsolidation [257]. Ketamine's dose-dependent dissociative and antipsychotic effects during treatment are transient, reverting to baseline within 2 hours and becoming less pronounced with repeated administrations [257]. Multiple investigations have shown that repeated ketamine leads to rapid and sustained improvement in depressive symptoms and PTSD, with the therapy deemed safe for repeated use [8, 257-259]. Thus, ketamine may be an effective approach to treating patients with comorbid PTSD and depression.

### 4.3. Alzheimer’s disease and dementia

In large-scale prospective epidemiological investigations, there exists a positive correlation between depression across different age stages (early, middle, or late) and increased susceptibility to the onset of AD [260]. It is of significance to note that a substantial hereditary link has been identified between depression and AD, with depression seemingly contributing causally to the progression of AD, driven in part by a total of 53 brain transcriptomes and proteins [260]. Following the amyloid hypothesis, generating excessive amyloid-beta (Aβ) through the upregulation of amyloid precursor protein or impaired clearance mechanisms leads to AD [261]. Moreover, heightened levels of soluble Aβ peptides in the cerebral region, especially Aβ_1-42_ oligomers, may also be associated with depression related to amyloid pathology [262]. The neuroactive properties of soluble Aβ_1-42_ directly interfere with the monoaminergic system and possess neuroregulatory effects [263]. Recently, evidence has surfaced indicating that Aβ disrupts serotoninergic functions (such as cortical 5-HT and NE reduction) and neurotrophic factor signaling, giving rise to depression [263]. This phenotype has been demonstrated to be potentially treatable with fluoxetine and ketamine in rodent models, which exert their effects through the augmentation of NE release [263, 264]. Notably, NE is a powerful neurotransmitter with neuroprotective properties against Aβ-induced neurotoxicity [264]. Consequently, the elevated levels of brain Aβ deposition in elderly individuals without cognitive impairment are positively correlated with escalated manifestations of anxiety-depressive symptomatology [265]. Further research indicates that cortical amyloid modulates the connection between worsening depressive symptoms and cognitive decline in the elderly population [266]. Thus, alterations in depressive states serve as potential indicators to target and potentially delay the onset of clinical symptoms associated with AD [266].

As formerly mentioned, heightened levels of glucocorticoids elicit detrimental effects on cerebral structure and function [210], particularly precipitating hippocampal morphological alterations that underlie the pathophysiological mechanisms of dementia and depression [267]. Literature has documented that glucocorticoids evoke a surge in APP expression, tau aggregation, and consequent modifications in tau phosphorylation states [267]. Positron emission tomography imaging findings about tau pathology in cognitively intact older individuals reveal an interconnection between depressive symptomatology and tau accumulation within regions susceptible to AD, namely the internal olfactory cortex and inferotemporal cortex [268]. Additionally, persistent elevation of glucocorticoid levels potentially fosters hippocampal cellular injury, apoptosis, hippocampal atrophy, and cognitive deterioration [269]. Comparative analysis of elderly subjects with and without depression unveils a reduction in hippocampal volume in individuals affected by late-life depression, prompting the proposition that chronic exposure to stress or depressive episodes instigates hippocampal diminution, thereby fostering the genesis of dementia [269].

Pathologies linked to the advancement of depression comprise astrocyte dysfunction and persistent microglia activation, which are also associated with AD [261]. As previously mentioned, microglia activation is more prevalent and progressive in depression than in untreated conditions [157]. Nevertheless, prolonged microglia activation is related to the progression of neurodegenerative diseases, including AD, with microglia-mediated innate immune responses significantly contributing to AD development [261]. Moreover, a lack of gut flora has shown an inclination towards modified microglia transcriptomics, which can be restored through microbial colonization [270]. Recently, research has revealed that transcriptomic changes in these microglial subpopulations are predominantly related to AD and depression [270]. Hence, prolonged microglia activation and gut flora-induced alterations in microglia transcriptomics could aid in understanding the correlation between depression and AD. Several research studies have documented that the levels of calcium homeostasis regulator protein 2 (Calhm2), a major ATP-release channel, are essential for neurological disorders like AD [271]. A deficiency of Calhm2 causes a noteworthy reduction in ATP release from astrocytes in the brain, leading to depressive-like behavior in mice [272]. Consequently, CALHM family proteins are essential for the pathogenesis of depression and AD [271, 272]. Recent studies have postulated that the CALHM2 V136G mutation is involved in the progression of depression and AD by regulating astrocyte ATP release [9]. The incidence of CALHM2 V136G mutation (rs232660) was conspicuously greater in AD patients compared to normal controls, disrupted ATP release from astrocytes and brain tissues, causing depressive-like behaviors and cognitive deterioration in adult and aged mice, respectively [9]. Interestingly, the administration of ATP rectified the depressive-like phenotype in Calhm2 V136G mice [9]. These analyses provide strong evidence supporting depression closely related to dementia, indicating that flawed CALHM function significantly contributes to the development of both conditions.

Presently, the FDA has solely approved acetylcholinesterase inhibitors and NMDA antagonists as the primary pharmacotherapies for AD, while the availability of medications targeting psychiatric symptoms is limited [273]. Ketamine, a non-selective NMDAR antagonist, engenders various pharmacological effects [274]. Remarkably, case reports already exist that illustrate the swift alleviation of severe refractory depression in AD patients following the administration of subcutaneous ketamine [275]. Furthermore, a solitary administration of ketamine considerably ameliorated depressive symptoms while preserving cognitive functions such as memory and learning [276]. The accumulation of current preclinical and clinical data provides support for the capacity of ketamine to antagonize NMDAR, diminish neuroinflammatory cytokines, and enhance neurocognitive functions in AD, increasing its neuroprotective efficacy [273]. Hence, the investigation of ketamine's potential to improve depressive symptoms in AD patients assumes paramount importance.

### 4.4. Parkinson’s disease

Depression is a prevalent comorbidity in individuals diagnosed with PD, exhibiting an approximate occurrence rate of 30-35% [10]. Moreover, PD patients exhibit a higher propensity for experiencing mood dysfunctions, such as anxiety, in comparison to the general population, with reported rates of clinically significant anxiety symptoms ranging between 20% and 52% [277]. Nevertheless, the etiology of anxiety in PD patients remains elusive, as it is uncertain whether neurochemical alterations inherent to the disease itself, psychological responses to the stresses associated with the condition, medication-related effects, or external factors unrelated to PD contribute to the anxiety levels observed [278]. The majority of PD patients exhibit coexisting anxiety and depression [277]. However, the mechanism of anxiety and depression in PD is still not fully understood, notwithstanding conventional dopaminergic models suggesting an association with dopaminergic dysfunction and changes in neuroanatomy that potentially impact emotional processing [279]. Currently, it is postulated that PD pathology may disrupt fear circuits through various mechanisms, leading to aberrant connectivity between anxiety-depression pathways and basal ganglia network activity [280, 281]. By utilizing optogenetic and chemical genetics techniques to modulate distinct subpopulations of the parafascicular thalamus (PF) projecting into the caudate-crustal nucleus (CPu), thalamus substrate nucleus (STN), and NAc, researchers have demonstrated that the PF→CPu and PF→STN circuits effectively restore motor-learning behaviors in a mouse model of PD [282]. Furthermore, activation of the PF→NAc circuit effectively ameliorates depressive-like symptoms [282]. Hence, therapeutic interventions targeting PF thalamic circuits may hold promise as a viable strategy for addressing non-motor deficits in PD [282].

Multiple investigations have reported that ketamine administration ameliorated suicidal ideation and severe depression in individuals with PD, while motor impairments and cognitive impairments are likewise resolved [283-285]. Ketamine averts neurotoxicity in the model of PD by stimulating BDNF-TrkB signaling [286]. It is noteworthy that refractory depression and levodopa-induced dyskinesia are characterized by hypersynchronous neural oscillations in PD [287, 288]. Prolonged exposure to ketamine elicits better coordination between low and high frequencies in the striatum and diminishes synchronization in the hippocampus [287]. Specifically, a low dose of ketamine enhances cortical-striatal cross-frequency coupling and hippocampal broadband gamma oscillations, reflecting the management of moods [287]. Collectively, ketamine may delay the progression of motor and non-motor symptoms in PD. Further studies are needed to examine ketamine's efficacy in relieving depressive symptoms in individuals with PD from a molecular mechanistic perspective.

### 4.5. Huntington’s disease

In HD, 33-69% of patients experience depressive symptoms, even in the early stages preceding typical motor symptoms [289]. Treating mood changes may decelerate disease progression and enhance the quality of life in individuals with HD [290]. However, the molecular mechanisms responsible for the depressive phenotype in HD have received less attention to date. Impaired Cyclin-dependent kinase 5 (CDK5) function is implicated in the pathophysiology of depressive behavior in HD, possibly through altered DARPP-32/β-adducin signaling and dendritic spine cytoskeleton disruption in the NAc of HD mice [11].

Although numerous drugs that modulate neurotransmitters are available for treating depression in HD, some of them may worsen chorea or fail to produce the desired beneficial effects [290]. These results emphasize the need for novel therapeutic strategies. Based on the findings mentioned earlier, targeting CDK5 activity might be beneficial as a fresh therapy to prevent or reduce depression in HD [11]. However, it is worthwhile noting that ketamine also modulates CDK5 activity [291]. Furthermore, in HD mice, astrocyte-mediated K^+^ homeostasis affects the buffering process of K^+^, leading to an accumulation of excessive K^+^ between cells, increasing the excitability of medium spiny neurons in the striatum and making them more prone to firing, which may be a primary cause of the characteristic choreiform symptoms in HD [292]. However, ketamine regulates K^+^ homeostasis through astrocyte-specific Kir4.1, inhibiting the ability of brain regions associated with aversive and negative emotions to erupt, thus alleviating depressive-like symptoms [135]. Therefore, targeting the Cdk5/DARPP-32/β-adducin signaling pathway or ion homeostasis may alleviate depressive symptoms in HD.

### 4.6. Type 2 Diabetes and insulin resistance

A causal positive correlation between T2D and depression has been observed [293]. Increasing evidence suggests that depression and T2D share common pathogenic mechanisms, mainly related to cytokine-mediated inflammatory responses triggered by hyperactivation of innate immunity, which may result in abnormalities in the regulation of the HPA axis [294]. For example, individuals with genetic susceptibility, exposure to maternal or fetal stress during pregnancy, low socioeconomic status, and unhealthy behaviors may experience dysregulation of the HPA axis, leading to disruptions in circadian rhythms and serving as a catalyst for innate inflammatory responses [294]. Imbalances in these biological pathways can simultaneously contribute to insulin resistance and depression [294]. In individuals with diabetes, depression heightens stress, leading to HPA axis hyperactivation, elevated cortisol levels, and reduced insulin sensitivity [12]. In addition, excessive pro-inflammatory cytokines cause the breakdown of TRP into neuroactive metabolites, leading to decreased serotonin levels and subsequent depressive symptoms [12]. Interestingly, before the introduction of antipsychotic medications, psychiatric patients admitted to hospitals exhibited abnormal glucose tolerance and impaired hepatic insulin sensitivity [295]. Notedly, the efficacy of current antipsychotic medications often correlates positively with the severity of their metabolic side effects, suggesting a connection between insulin signaling disruptions and their therapeutic mechanism [295].

Insulin signaling facilitates neuroplasticity, neurogenesis, and neuroprotection and effectively harnesses metabolic mechanisms for bioenergetic production [296]. Notably, animal models with brain insulin receptor knockout manifest depressive and anxiety-like phenotypes alongside hypothalamic-function-associated metabolic traits, propounding that the insulin signaling pathway within the brain governs not merely peripheral metabolism but also emotional cognition-related behaviors [297]. Likewise, insulin signaling exhibits critical significance in astrocytes [298]. The emergence of evidence has revealed that the modulation of dopamine release via the astrocyte insulin signaling pathway, which is achieved by the regulation of ATP release, ultimately exerts depressive-anxiety-like behavioral patterns [298]. Consequently, targeting the insulin signaling pathway could potentially mitigate diabetic depression [299]. Moreover, lithium augmentation of ketamine ameliorated antidepressant-like responses to stress, peripheral insulin efflux, and region-specific PFC insulin signaling [299]. Additionally, perturbations in IGF-1 levels are linked to impaired glucose tolerance and susceptibility to T2D, emphasizing the importance of maintaining IGF-1 homeostasis within the brain [300]. Interestingly, the release of IGF-1 in the mPFC mediates the rapid and enduring antidepressant-like effects of ketamine [301]. Therefore, conducting further investigations to establish the potential relationship between IGF-1 and ketamine-resistant depressive behavior in T2D is warranted.

IL-1β is a pivotal inflammatory mediator implicated in diabetes mellitus and depression, associated with compromised insulin secretion and various depressive symptom mechanisms [302, 303]. In fact, chronic hyperglycemia may induce the secretion of IL-Ι β via the activation of the NLRP3 inflammasome facilitated by the production of ROS and thioredoxin-interacting protein (TXNIP) [304]. Recent investigations have underscored that hyperglycemia stimulates inflammatory factors to activate microglial NLRP3 in hippocamp, upregulating P2X7R and enhancing ROS production and TXNIP expression, all of which likely mediate the emergence of depression in diabetic mice model [302]. Many investigations have demonstrated that ketamine administration exerts a robust antidepressant effect by downregulating inflammatory cytokine mediators, including IL-1β, within the hippocampal region [181]. Hence, it can be surmised that the mitigation of diabetic depressive symptoms after ketamine administration may be attributed to the consequential downregulation of IL-1β. In addition, evidence indicates that the association between T2D and depression may be influenced by BMI, highlighting the importance of maintaining a healthy weight in the management of comorbid depression and T2D [293]. Nonetheless, in contrast to traditional antidepressants, ketamine exhibits greater efficacy in individuals with elevated BMI, particularly those with pronounced abdominal obesity [305]. Therefore, the ability of ketamine to elicit a favorable response in this subgroup may confer a potential advantage.

### 4.7. Multiple Sclerosis

MS is a chronic demyelinating autoimmune disease that significantly impacts daily activities and imposes a substantial economic burden on patients, caregivers, and the healthcare system [306]. Psychiatric comorbidities, including depression and anxiety disorders, are frequently encountered in individuals afflicted with MS [13]. Extensive investigations have shown that depression in MS patients is intimately linked to increased susceptibility to vascular disease and mortality [307]. The prevalence of suicide rates in MS patients has been reported to soar as high as 22.1%, rendering it twice as prevalent as in the general population [308]. Concurrently, depressive symptoms are typically entwined with a progressive course of MS, and early depressive symptoms are predictive of disability accumulation, arguably arising as a consequence rather than a causative factor [309]. The etiology of depression in MS is multifaceted, ranging from genetic predisposition, immune dysregulation, and structural anomalies to functional brain injuries [13]. Remarkably, aberrant communications between integral anatomical regions implicated in task-specific emotion regulation, such as the amygdala and the ventral lateral prefrontal cortex, have been noted in MS patients, even before the onset of depression [310].

Given the social and economic burden of comorbid depression in MS, there is a compelling need to develop pharmacological interventions to impede or delay disease progression and severity [311, 312]. Previous studies have demonstrated the efficacy of Clemastine in facilitating the regeneration of myelin in MS, but not within the context of depression [98]. Conversely, ketamine has been found to effectively counteract myelin impairment while concurrently exerting antidepressant properties [108]. Therefore, ketamine may represent an alternative treatment for refractory depression in MS patients and could have a unique therapeutic potential in slowing demyelination and promoting myelin regeneration [109, 313, 314]. Cuprizone (CPZ), a selectively sensitive copper-chelating agent, has been employed to elicit the onset of noxious demyelination like that within MS lesions [315]. The intermittent administration of ketamine in the brains of CPZ-treated mice has displayed substantial improvements in demyelination and discernible alterations in the gut microbiota composition [109]. Additionally, other evidence has corroborated that ketamine enhances the synthesis of myelin basic protein and neurofilament heavy-chain protein in CPZ-treated rats, contributing to new dendritic spines and myelination [314].

On the other side, persistent weariness represents a prevalent and incapacitating symptom in MS patients, particularly those with comorbid anxiety disorders and depression, who suffer from more pronounced and protracted chronic fatigue [316]. Unfortunately, evidence suggests that amantadine, modafinil, and methylphenidate have evinced no discernable superiority over placebo in decreasing fatigue in MS patients [312, 317]. By contrast, low doses of ketamine have rapidly lessened the severity of fatigue, with its anti-fatigue effects mediated by the antidepressant properties of ketamine [318]. Similarly, double-blind, randomized, placebo-controlled trials have indicated that low-dose ketamine significantly ameliorates fatigue severity in MS patients [311, 312]. Nevertheless, additional investigations that adopt larger randomized trials may shed light on the effectiveness of ketamine in MS patients with neuropsychiatric symptoms.

### 4.8. Osteoporosis

Previous investigations have delineated various biological mechanisms that underlie the coexistence of depression and osteoporosis, encompassing the depletion of essential factors like vitamin D, estrogen, and testosterone, inflammatory responses, perturbations in the HPA axis, and elevated levels of cortisol in plasma, all of which culminate in reduced bone mineral density (BMD) during depressive states [319]. Emerging evidence indicates the involvement of GABAergic neural circuitry in the ventromedial hypothalamus (VMH) in stress-induced bone loss [320]. Chronic anxiety activates somatostatin neurons in the BNST, reducing SF-1 neuron activity in the VMH and inhibiting the nucleus tractus solitarius excitatory [320]. Presently, the current association between widely used osteoporosis medications and the heightened occurrence of depression and anxiety [321]necessitates the development of innovative antidepressants that exhibit positive effects on osteoporosis.

The osteoprotegerin (OPG)-RANK-RANKL system, or osteopontin (OPN), is a predictive indicator of bone inflammation in the skeletal abnormalities associated with deprssion [322]. In particular, the receptor activator of nuclear factor-κB ligand (RANKL) serves as a differentiation factor for osteoclasts and contributes to reduced BMD, while OPG augments BMD [323]. In addition, OPN acts as a bone glue by linking the matrix components of bone tissue, playing a pivotal role in bone strength and fracture resistance [323]. Following the administration of ketamine, remarkably increased ratios of OPG/RANKL, elevated plasma OPN levels, and reduced RANKL levels, indicating the potential of ketamine in enhancing BMD [322]. Similar outcomes were also noticed in an animal model of postmenopausal osteoporosis, such as ovariectomized (OVX) mice [324]. Subsequent investigations revealed that ketamine triggers gut microbiota's anti-inflammatory properties, which may contribute to cortico-BMD and total BMD improvement in OVX mice [325]. Consequently, ketamine emerges as a promising regimen for managing the perturbations in bone metabolism that often coexist with depression.

### 4.9. Inflammatory bowel disease

IBD is a persistent ailment characterized by a notable relapse rate, encompassing Crohn's disease and ulcerative colitis [326]. Comorbidities such as depression and anxiety are frequently observed in individuals with IBD [327]. However, the prevalence rates of these comorbidities vary across investigations due to differences in research populations and assessment tools for depression and anxiety [327]. The current understanding of the relationship between depression and IBD remains ambiguous. However, individuals with prior gastrointestinal symptoms and depression are more prone to developing Crohn's disease or ulcerative colitis [328]. Similarly, experimental models employing dextrose sodium sulfate (DSS) colitis have revealed a correlation with heightened anxiety and depression-like behaviors [14]. Notably, a systemic link exists between the gut and the brain facilitated by the gut vascular barrier (GVB) and plexus vascular barrier (PVB), respectively [329]. In intestinal inflammation, disruption of the GVB coincides with bacterial product translocation, ultimately leading to immune cell recruitment and inflammation propagation to the brain [329]. On the other hand, the inflammatory response induced the closure of the PVB after the opening of the GVB, which may detrimentally impact inter-organ communication and, ultimately, correlate with mental deficits [329]. Consequently, psychiatric symptoms associated with IBD could be attributed to dysregulation of the gut-brain-vascular axis. Moreover, alterations in the abundance of gut microbiota, particularly *Deltaproteobacteria*, have been demonstrated in mice exhibiting depressive-like behaviors [234]. Thus, the upregulation of *Deltaproteobacteria* levels may contribute to the depressive-like phenotype observed through inflammatory responses [330]. Consequently, the reduction in *Deltaproteobacteria* levels noticed in ketamine-treated mice with depressive-like behaviors may offer a partial explanation for its antidepressant effects in patients with UC [234].

Experimental colitis is characterized by central inflammation and cellular activation, underlying the emergence of symptoms related to depression and anxiety [14]. Chronic stress induced the prolonged elevation of glucocorticoid levels, generating an inflammatory subset of enteric glial cells and promoting inflammation mediated by monocytes and TNF [331]. Furthermore, glucocorticoids contribute to transcriptional immaturity of enteric neurons, deficiency of acetylcholine, and dysmotility through the action of TGF-β2 [331]. Hence, the enteric nervous system mediates the exacerbating effects of chronic stress on intestinal inflammation, emphasizing the potential value of stress management for IBD care. Following DSS-induced colitis, peripheral inflammatory responses disrupt the balance of microglial cell-intrinsic immune receptors TREM-1 and TREM-2 in the ACC, driving abnormalities in glutamatergic neuron modulation, ultimately contributing to visceral hypersensitivity and depressive-like behaviors [332]. Similarly, the upregulation of lipocalcin 2 (Lcn2), a vital regulator of the inflammatory response in the DSS model, causes the loss of dendritic spines and secreted proteins, resulting in multiple chemokines expression [333]. Subsequently, the activation of microglia contributed to increased permeability of the blood-brain barrier and inducted depression-like behaviors [333].

Notably, the occurrence of microbial dysregulation is a prevalent characteristic observed in both IBD and depression [334, 335]. Studies have shown that Estrogen receptor β plays a role in mediating colitis and anxiety-depression-like behaviors by disrupting neural processing within the gut-brain axis [335]. Moreover, clinical investigations have demonstrated the association between TRP metabolism and the severity of IBD [336]. Furthermore, the disturbance in TRP metabolites, including serotonin, QUIN, and KYNA, has been linked to depressive behavior [158]. During IBD, the persistence of chronic inflammation leads to excessive activation of IDOs, resulting in the heightened degradation of TRP into KYN [337]. Consequently, KYN across the blood-brain barrier, leading to an excess of QUIN, may contribute to depressive behaviors [337].

Overall, the relationship between IBD and depression appears to be bidirectional [327]. Notably, ketamine shows beneficial effects in a DSS-induced colitis model, suggesting its potential as a therapeutic agent for IBD [338]. Consequently, conducting further double-blind, placebo-controlled studies on patients with IBD, with or without comorbid depression, would be highly valuable [86].

## 5. Conclusion and outlook

Depression, a complex psychiatric disorder, is influenced by both significant genetic and non-genetic predisposing factors. Despite decades of research on animal models and postmortem tissues from individuals with depression, only a few novel therapeutic approaches have emerged. Fortunately, multiple tools such as optogenetic, genetic, and epigenetic have greatly expanded the opportunity better to understand the cellular and circuitry mechanisms underlying depressive-like behaviors. Furthermore, depression is a risk factor for various neurological disorders, though its etiology among individuals with these disorders is complex. The rapid antidepressant effects of ketamine in depression patients have revitalized both clinical and preclinical neuropsychiatry. In addition to NMDARs, ketamine and its enantiomers also act as partial agonists of μ and κ-opioid receptors (MORs and KORs, respectively), but they do not seem to bind to δ-opioid receptors (DORs) [339]. It has been demonstrated that selective MORs and KORs antagonist block the opioid receptor antagonistic effects of ketamine, impairing behavioral responses in rodents and attenuating antidepressant effects in patients with depressive disorders [340]. Therefore, other receptors such as MORs and KORs also appear to be involved in the antidepressant effects of ketamine, but their contributions to ketamine's neuronal effects remain to be further elucidated [339, 340].

**Figure 6.**
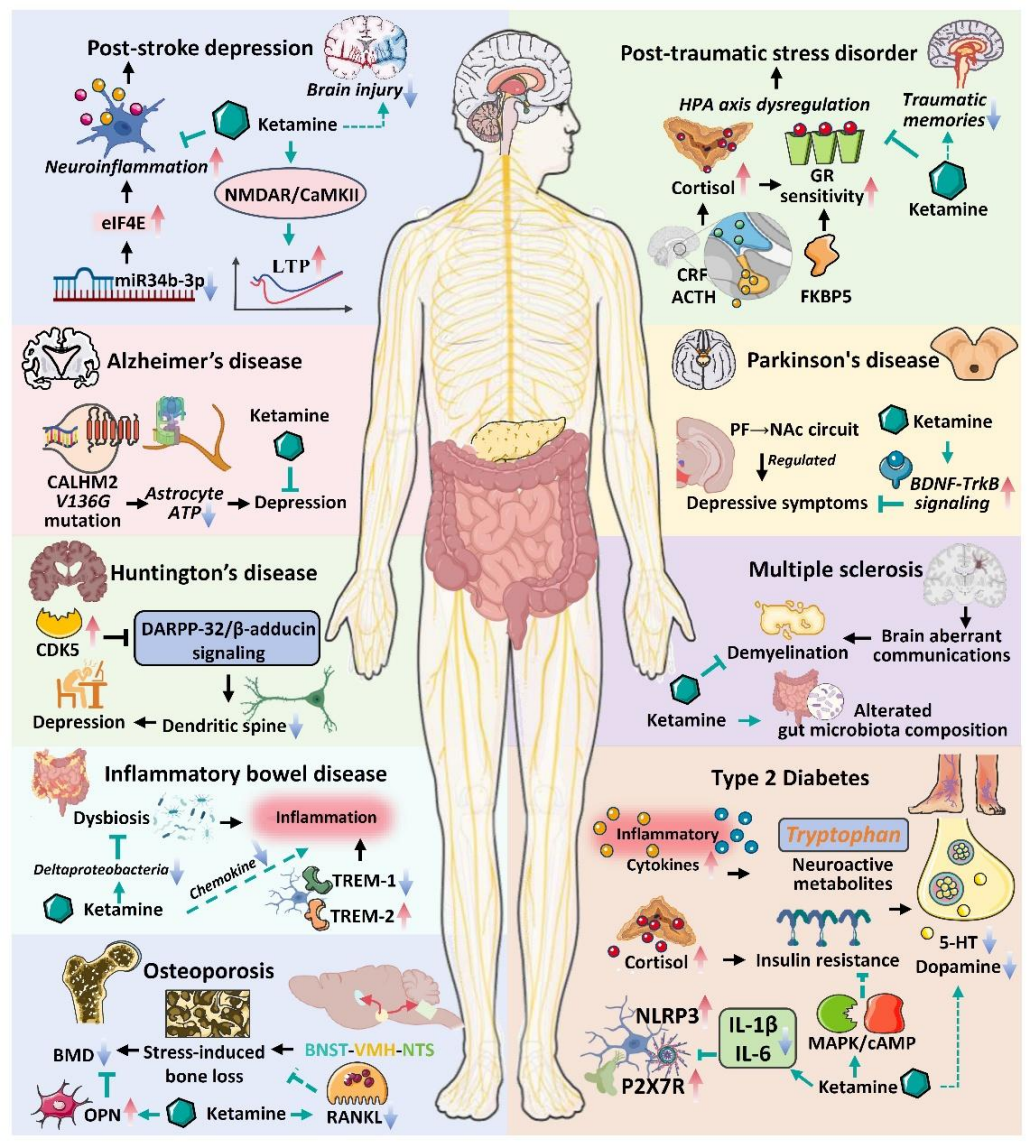
**The potential of ketamine in stress-related psychiatric disorders**. Ketamine, with its beneficial effects, has been shown to improve anxiety and depressive-like behaviors as well as pathological features of stroke, PTSD, cognitive impairment in mental illnesses, neurodegenerative diseases, osteoporosis, IBD, and T2D. For instance, (1) post-ischemic brain injury and behavioral deficits caused by ischemia can be effectively improved by administering ketamine after mouse middle cerebral artery occlusion; (2) ketamine reduces the negative impact of traumatic memories on PTSD by interfering with memory reconsolidation; (3) administration of subcutaneous ketamine rapidly alleviates severe treatment-resistant depression in patients with AD, while preserving cognitive functions such as memory and learning; (4) in a model of PD, ketamine mitigates the neurotoxicity of MPTP on dopaminergic neurons in the substantia nigra; (5) targeting the Cdk5/DARPP-32/β-adducin signaling pathway helps alleviate depressive symptoms in HD; (6) ketamine improves gut microbiota composition and promotes remyelination in an animal model of MS; (7) continuous administration of ketamine in DSS-induced IBD mouse model significantly improves microbial dysbiosis and reduces inflammation; (8) ketamine alleviates symptoms of osteoporosis by inhibiting plasma RANKL; (9) ketamine improves depressive symptoms in T2D by enhancing insulin signaling and reducing inflammation.

The main molecular mechanisms and targets underlying ketamine's antidepressant effects remain unclear, necessitating further research using novel techniques to fully elucidate its action and identify new intervention targets. Concurrently, clinical studies targeting ketamine have demonstrated its potent antidepressant effects and lower adverse reaction rates, paving the way for the development of safer and more effective antidepressant therapies for severe depression patients. It is noteworthy that, in addition to focusing on targets, future studies should also identify the biomarkers for ketamine responsiveness and explore its long-term effects. A deeper understanding of the mode of action between ketamine and targets, identifying biomarkers for ketamine responsiveness, exploring its long-term effects, and the pharmaceutical and pharmacokinetic properties of the drugs would facilitate the translational application of ketamine in depression and other psychological diseases. In addition, ketamine may also have broad application prospects in the treatment of stroke; PTSD; cognitive impairments in psychiatric disorders; neurodegenerative diseases; osteoporosis; IBD; and T2D; among other conditions, suggesting the repurposing of ketamine in the treatment of depression and depression-related disorders (Fig. 6).
